# The in vivo structure of biological membranes and evidence for lipid domains

**DOI:** 10.1371/journal.pbio.2002214

**Published:** 2017-05-23

**Authors:** Jonathan D. Nickels, Sneha Chatterjee, Christopher B. Stanley, Shuo Qian, Xiaolin Cheng, Dean A. A. Myles, Robert F. Standaert, James G. Elkins, John Katsaras

**Affiliations:** 1 Shull Wollan Center—A Joint Institute for Neutron Sciences, Oak Ridge National Laboratory, Oak Ridge, Tennessee, United States of America; 2 Biology and Soft Matter Division, Oak Ridge National Laboratory, Oak Ridge, Tennessee, United States of America; 3 Department of Physics and Astronomy, University of Tennessee, Knoxville, Tennessee, United States of America; 4 Biosciences Division, Oak Ridge National Laboratory, Oak Ridge, Tennessee, United States of America; 5 Center for Molecular Biophysics, Oak Ridge National Laboratory, Oak Ridge, Tennessee, United States of America; 6 Department of Biochemistry & Cellular and Molecular Biology, University of Tennessee, Knoxville, Tennessee, United States of America; 7 Department of Microbiology, University of Tennessee, Knoxville, Tennessee, United States of America; Centro Nacional de Biotecnologia, Spain

## Abstract

Examining the fundamental structure and processes of living cells at the nanoscale poses a unique analytical challenge, as cells are dynamic, chemically diverse, and fragile. A case in point is the cell membrane, which is too small to be seen directly with optical microscopy and provides little observational contrast for other methods. As a consequence, nanoscale characterization of the membrane has been performed ex vivo or in the presence of exogenous labels used to enhance contrast and impart specificity. Here, we introduce an isotopic labeling strategy in the gram-positive bacterium *Bacillus subtilis* to investigate the nanoscale structure and organization of its plasma membrane in vivo. Through genetic and chemical manipulation of the organism, we labeled the cell and its membrane independently with specific amounts of hydrogen (H) and deuterium (D). These isotopes have different neutron scattering properties without altering the chemical composition of the cells. From neutron scattering spectra, we confirmed that the *B*. *subtilis* cell membrane is lamellar and determined that its average hydrophobic thickness is 24.3 ± 0.9 Ångstroms (Å). Furthermore, by creating neutron contrast within the plane of the membrane using a mixture of H- and D-fatty acids, we detected lateral features smaller than 40 nm that are consistent with the notion of lipid rafts. These experiments—performed under biologically relevant conditions—answer long-standing questions in membrane biology and illustrate a fundamentally new approach for systematic in vivo investigations of cell membrane structure.

## Author summary

The structure and organization of the cell membrane are central to many biological functions, and although they have been extensively studied, there is still much that we don’t understand. A wealth of detailed information has been obtained from studies of model lipid membranes. However, these systems are often highly simplified in composition and always lack the active processes found in cells. The major obstacle to studying membranes in vivo in living cells has been that high-resolution physical methods used to investigate membrane structure are not compatible with living organisms. To overcome this obstacle, we employed a new in vivo approach that allows membrane structure to be observed directly in the gram-positive bacterium *B*. *subtilis*. This approach relies on tuning the isotopic content of hydrogen within the membrane, and other parts of the bacterium, to generate neutron scattering spectra exclusively from the membrane. Using this approach, we confirmed that the structure of the *B*. *subtilis* membrane is lamellar and has an average hydrophobic thickness of 23.9 ± 0.9 Ångstroms (Å). We were also able to observe nanoscopic lateral membrane structures, consistent with the notion of lipid rafts. This experimental approach will allow for a wide range of future structural studies of the cell membrane (and possibly other classes of biomolecules) without the need for extrinsic probes or labels. In this way, it fundamentally changes the scope of nanoscale structural questions that can be addressed in vivo.

## Introduction

Because of the inherent complexity of living cells, high-resolution analytical methods are generally used ex vivo, and many depend on labels to differentiate the species of interest. In the case of cellular membranes, their nanoscopic structure (ultrastructure) has been elucidated primarily through electron microscopy (EM) with heavy-atom labels[1], supplemented with in vitro biophysical studies of model membranes.[2–12] In vivo membrane studies at lower resolution, notably with fluorescence microscopy, have provided additional details of lateral structure as well as important information on diffusion and other dynamic processes.[13] Through these techniques, a vivid picture of the membrane has emerged.[14–19] Nonetheless, they have significant limitations, and fundamental questions about the ultrastructure of the membrane in vivo remain unresolved.[20]

For example, membrane hydrophobic thickness is a basic structural parameter with implications for membrane transport, as well as membrane protein folding and function. Yet the hydrophobic thickness of membranes has never been determined in vivo. Another outstanding question concerns the existence of nanoscopic domains, or lipid rafts. The lipid raft hypothesis[21] invokes lateral organization of membrane lipids and proteins into distinct domains in the plane of the membrane to facilitate the assembly and regulation of multi-molecular complexes. This hypothesis provides a compelling rationale for numerous observations relating to membrane trafficking, endocytosis, signal transduction, and other processes.[22–25] However, evidence for lipid domains has been largely inferential, and it is now widely believed that these domains are nanoscopic as well as transient[26,27], making them difficult to detect. New methods to interrogate lateral structure in vivo—ideally without the use of extrinsic probes—would help us to understand the role of lateral heterogeneity in the many cellular processes where it has been implicated.

In this context, small-angle neutron scattering (SANS) has emerged as a uniquely powerful tool for the study of lipid bilayer structure in vitro. Neutrons have wavelengths on the order of Ångstroms (Å) and are thus inherently nanoscopic probes, well-matched to the dimensions of membrane structure. Using appropriately designed experiments, both transverse (normal to the membrane plane)[7] and lateral (within the membrane plane)[28,29] structure can be accurately determined. Importantly, neutron-scattering techniques do not require extrinsic molecules or heavy atoms as labels and rely instead on hydrogen (H)/deuterium (D) isotopic substitution. Cold and thermal neutrons are also nondestructive[30–32], making them ideal for the study of living systems. However, applications of powerful neutron-based ultrastructural methods have been restricted to in vitro membrane models due to the complex scattering signal arising from cells as a result of their diverse biomolecular composition.

Scattering and imaging experiments require that the feature(s) of interest have an observable signal, contrasted from the background. Where contrast is insufficient in the native system, it must be imparted through the use of labels. In the case of fluorescence imaging, contrast arises from the distinctive excitation and emission properties of native, or more commonly, introduced fluorophores. With X-rays and electrons, scattering contrast arises from differences in electron density, which scale with atomic number. Neutrons, on the other hand, are scattered by atomic nuclei, and the key contrast parameter—analogous to electron density—is the scattering length density (*ρ*). Neutron scattering lengths (*b*) are unrelated to atomic number and, in fact, vary among isotopes of the same element (S1 Fig). Most importantly, the scattering lengths of hydrogen (*b*_H_ = −3.74 fm) and deuterium (*b*_D_ = 6.67 fm) are substantially different.[31] Thus, neutron contrast is unique in that it can be varied in hydrogen-rich biological systems with H/D isotopic labels, as opposed to the heavy-atom labels used in X-ray and electron scattering, or fluorescent labels used in microscopy.

**Fig 1:**
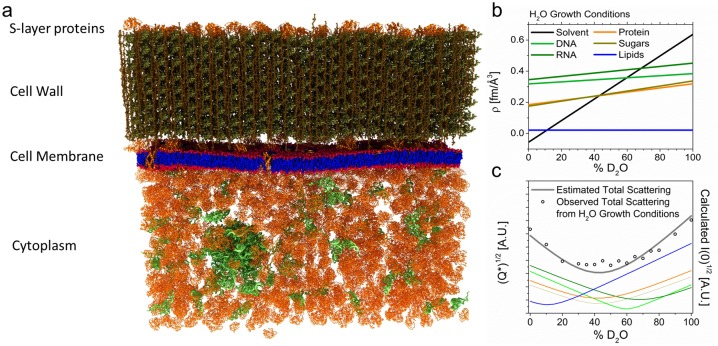
Envelope structure and scattering properties of *B*. *subtilis*. (a) Representation of the cell wall, the membrane and a portion of the cytoplasm. (b) Neutron scattering length densities, *ρ*, of the different unlabeled biomolecules as a function of percent D_2_O in the aqueous medium. Line colors correspond to (a). Sloped lines are the result of partial deuteration via exchange of NH, OH, and SH hydrogens with increasingly deuterated water. Where the line for each type of biomolecule crosses the black water line, e.g., at approximately 42% D_2_O for protein (orange line), the class of biomolecule is contrast-matched and is effectively invisible to neutrons. (c) Total neutron scattering from cells grown in ordinary hydrogen (H) medium and resuspended in phosphate buffered saline (PBS) at different D_2_O concentrations (see S1 Data). The estimated intensity *I*(0) is shown with the observed intensity, expressed as the Porod invariant Q*, from small-angle neutron scattering (SANS) experiments. *I*(0) was calculated using the *ρ* values in (b) and the biomolecular composition of *B*. *subtilis* (Tables A-E in S1 Text). The contribution of individual species is shown using the same color scheme as in (b). Intensity scales with Δ*ρ*^2^ and is plotted as its square root to maintain proportionality in the ordinates between (b) and (c).

Different classes of biomolecules (i.e., proteins, lipids, carbohydrates, and nucleic acids) have different elemental compositions, which gives each class a different value of *ρ* (Fig 1a and 1b; Table A in S1 Text). Multicomponent systems can be designed such that 2 or more components have the same *ρ*, for example, protein in approximately 42% D_2_O/H_2_O. In this case, the protein and solvent are said to be contrast-matched, and the protein does not generate a distinct scattering signal—i.e., it is effectively invisible to neutrons.[30] If a third component is introduced having a different *ρ* (e.g., DNA), it then becomes the only contributor to the net scattering. Through judicious H/D-labeling of the sample and the aqueous medium, *ρ* for the different biomolecules can be tuned to enhance or attenuate their scattering. Thus, contrast can be varied without changing the chemical composition of the system.

In this report, we describe a chemical–biological approach to controlling neutron contrast variation in vivo and its application to the study of cell membrane ultrastructure. As a platform, we chose the gram-positive bacterium *B*. *subtilis*. This organism has a number of attractive features, such as being genetically tractable, having a well-characterized lipid metabolism, and growing readily in deuterated media. Importantly, it has a single membrane and uses only saturated fatty acids (FAs) that can be prepared readily in deuterated form to allow tuning of membrane contrast. After suppressing cellular contrast through global D-labeling (i.e., replacing most of the H with D throughout the cell), we selectively reintroduced contrast to the membrane by supplying H-FAs, which the cell incorporated. In this way, we were able to isolate the SANS spectrum of the membrane, which showed it to be a lamellar structure with a hydrophobic thickness of 24.3± 0.9 Å. A modification of the contrast-labeling scheme led to the observation of nanoscopic lipid structures of <40 nm within the plane of the cell membrane, providing experimental evidence for the existence of nanoscopic lipid domains (lipid rafts) in an active biological membrane.

## Results

### Total cellular neutron contrast matching strategy for *B*. *subtilis*

The neutron scattering signal from a cell reflects the sum of contributions from all cellular components, here, taken to be water, protein, RNA, DNA, carbohydrate, and lipid (Fig 1a). Each of these has a characteristic *ρ* (Fig 1b), and from their relative abundances in *B*. *subtilis*,[33] we estimated the total scattering from the organism as a function of D_2_O concentration in the aqueous environment. Water accounts for approximately 85% of the cellular volume and exchanges rapidly across the membrane. Therefore, immersion of cells in deuterated buffer changes the neutron contrast, ergo the net scattering, with the relative contributions of the different molecular species varying as a function of D_2_O concentration (Fig 1c; S2 Fig).

We then measured the total scattering from *B*. *subtilis* cultured in standard M9 minimal medium (H-M9) and resuspended in phosphate buffered saline (PBS) at different D_2_O concentrations. The observed scattering corresponded closely to predictions of the simple compositional model, as shown in Fig 1c. Note that the cells scatter strongly over the entire range of percent D_2_O, and the total signal at any concentration of D_2_O reflects contributions from the multiple classes of biomolecules present in the cell. Structural analysis based on the overlapping signals is impractical, if not impossible.

**Fig 2:**
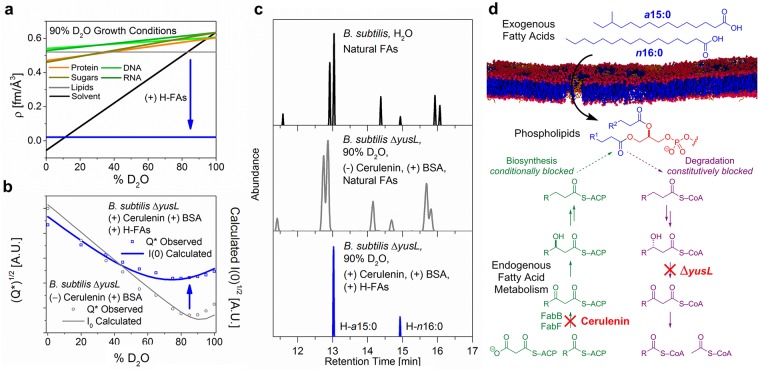
Growth conditions to control membrane composition and neutron contrast. (a) Scattering length densities, *ρ*, for the different classes of biomolecules in *B*. *subtilis* cultured in 90% D_2_O with hydrogen (H)-glucose. Compared to Fig 1b, there is a clear shift of *ρ* to higher values for all classes of biomolecules as a result of deuteration, converging in the range 90%–100% D_2_O. Incorporation of exogenous H-fatty acids (FAs; solid blue line) generates strong neutron contrast (blue arrow down). (b) Total neutron scattering from cells grown in deuterated medium and resuspended in phosphate buffered saline (PBS) at different D_2_O concentrations. The observed scattered intensity Q* is shown along with the calculated intensities *I*(0) for Δ*yusL* cells grown in the absence (open circles) or presence (open squares) of cerulenin and H-FAs. The difference at 85% D_2_O (blue up arrow) results from the strong contrast between H-FAs and the rest of the cell and buffer. (c) Cellular lipids were extracted, then methanolyzed to liberate FAs as methyl esters for analysis by gas chromatography/mass spectrometry (S3 Fig). (top) *B*. *subtilis* membranes have a mixture of 7 linear and branched saturated FAs.[34] (middle) Growth of cells in deuterated medium does not significantly alter FA composition (S4 Fig and Table B in S1 Text). Peaks shift because deuteration reduces retention on the column and broaden due to the presence of multiple isotopomers, averaging approximately 70% deuterium (D). (bottom) Cerulenin-treated Δ*yusL* cells rescued by the addition of 2 native FAs, H-anteiso-pentadecanoic acid (a15:0) and H-normal-hexadecanoic acid (n16:0), incorporate only these 2 FAs in their membranes. See S2 Data. (d) FA degradation via β-oxidation was suppressed by deletion of the *yusL* (*fadN*) gene (locus BSU32840), and FA biosynthesis was blocked conditionally by the addition of cerulenin to the medium. Exogenous FAs rescue growth and become incorporated into membrane phospholipids.

To isolate a meaningful scattering signal from the membrane, it was first necessary to suppress neutron contrast from the entire cell by manipulating its H/D composition. This objective was achieved by culturing *B*. *subtilis* in deuterium-enriched M9 minimal medium (D-M9, prepared in 90% D_2_O with H-glucose as the carbon source). Due to metabolic H/D exchange, deuterium from the growth medium becomes permanently incorporated into the carbon skeletons of biosynthetic molecules. Overall, skeletal deuteration was approximately 70% (S4 Fig, Tables B and C in S1 Text), which was predicted to create a near-contrast matched condition for all biomolecules when the cells were immersed in approximately 85% D_2_O buffer (Fig 2a). This expectation was borne out in the scattering experiment, where a strong reduction in the total scattering was observed at approximately 85% D_2_O (Fig 2b, open circles; S2 Fig).

### Incorporation of H/D-labeled FAs into the cellular membrane

With contrast suppressed, the next step was to reintroduce contrast specifically into the membrane by providing exogenous FAs for incorporation into the membrane phospholipids. Membrane FAs, after conversion to FA methyl esters (FAMEs), are readily analyzed for structure and isotope substitution by gas chromatography/mass spectrometry (GC/MS). As shown in Fig 2c (top), *B*. *subtilis* uses a mixture of 7 main FAs, all of which are saturated (have no double bonds), and all but one of which are branched.[34,35] Initial supplementation experiments with wild-type *B*. *subtilis* showed that exogenous FAs were not incorporated into the membrane lipids intact, so the native pathways for both catabolism and anabolism of FAs had to be blocked (Fig 2d).

Catabolism was blocked genetically by the deletion of *yusL*, the gene encoding a critical enzyme in β-oxidation, enoyl-CoA hydratase.[36] Anabolism was blocked chemically using cerulenin, an irreversible inhibitor of β-ketoacyl-ACP synthase that suppresses de novo FA biosynthesis.[37] This combination resulted in conditional FA-dependent growth (demonstrated in S5 Fig). Cerulenin-induced growth inhibition can be rescued with a mixture of just 2 of the native complements of FAs—palmitic acid (normal-hexadecanoic acid [n16:0]) and 12-methyltetradecanoic acid (anteiso-pentadecanoic acid [a15:0]).[38] These 2 constitute a minimal set of 1 high-melting (n16:0) and 1 low-melting (a15:0) FA, with which the cell can regulate the fluidity and structure of its membrane. Unlabeled (H) and perdeuterated (D) forms of these 2 FAs were then used in various mixtures to tune neutron contrast in the membrane.

Growth of *B*. *subtilis* Δ*yusL* in D-M9 medium did not alter the FA composition of the membrane (Fig 2c, upper and middle panels; see S4 Fig for peak assignments and Table B in S1 Text for peak areas and deuteration analysis). However, when cerulenin-treated Δ*yusL* cells were grown in the same D-M9 medium, supplemented with H-n16:0 and H-a15:0, their membrane FAs were found to consist exclusively of these 2 FAs, with no deuterium incorporated from the medium (Fig 2c, bottom panel). The total neutron scattering from these cells showed a large increase in scattering at 85% D_2_O (marked by the blue arrow in Fig 2b), which can be attributed entirely to the H-FAs incorporated into the membrane phospholipids.

### SANS measurements to determine hydrophobic membrane thickness

**Fig 3:**
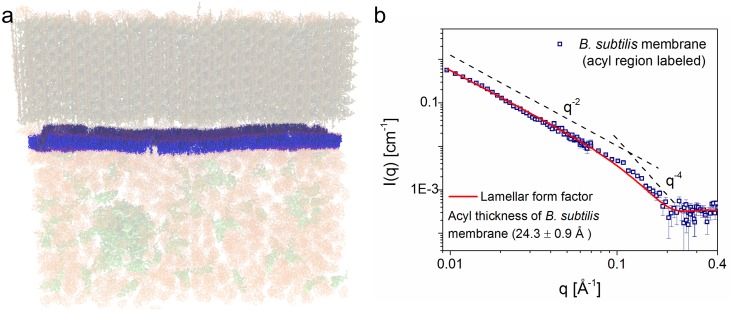
Contrast variation small-angle neutron scattering (SANS) reveals the structure of the *B*. *subtilis* membrane. (a) Schematic of the hydrogen (H)-labeled membrane as seen by neutrons. The cell wall and cytoplasmic contents are contrast-matched at 85% D_2_O, hence invisible to neutrons, while the membrane with incorporated H-fatty acids (FAs) stands out in strong contrast. (b) SANS data plotted as scattered intensity, *I(q)*, as a function of scattering wavevector, *q* (Å^−1^), from the *B*. *subtilis* membrane. Error bars correspond to ± *σ*. The experimental data are shown superimposed with the best-fit (red solid line) using a lamellar form factor,[45] revealing an average hydrophobic thickness of 24.3 ± 0.9 Å, consistent with a fluid-phase lipid bilayer (see S3 Data). Gas chromatography/mass spectrometry (GC/MS) analysis of the membrane FAs is shown in Fig 2c with peak integrals in Table E in S1 Text.

Having imparted contrast to the membrane (Fig 3a), we used SANS to determine the transverse membrane structure by recording the scattered intensity, *I(q)*, as a function of the scattering wavevector, *q*. Cells for this experiment were cultured as described above in D-M9 medium, supplemented with cerulenin and H-n16:0 and H-a15:0 to label the membrane, then transferred to 85% D_2_O buffer. Because these are more time-consuming experiments, the cells were resuspended in an 85% D_2_O buffer supplemented with glucose, Mg^2+^, and cerulenin. These additives prevent autolysis and preserve the membrane potential,[39,40] such that cells in suspension remained >90% viable over a period of 4 h at 25°C, as determined by direct cell counts, optical density measurements, and live/dead staining (S6 and S7 Figs). The residual background was recorded using cerulenin-treated Δ*yusL* cells, which were fed a mixture of FAs contrast-matched to 85% D_2_O (a15:0 and n16:0, each 30% H and 70% D), and which do not contribute substantially to the net neutron scattering signal.

In SANS, the shape of the *I(q)* versus *q* data describes the structure of the sample on the order of Ångstroms to approximately 100 nm. Formally this data is modeled using a lamellar form factor representing the acyl core of the bilayer, with the 2 identical head group regions on either side, contrast-matched to the scattering density of the surrounding cellular environment. The lamellar form factor is characterized by a *q*^−2^ dependence at low-*q*, arising from the 2D membrane surface of the entire bacterium, transitioning to a *q*^−4^ dependence typical of 3D objects with smooth surfaces and sharp interfaces—more detail is available in the literature[41–44] and the **materials and methods**.

Subtraction of the background from the sample scattering revealed a pure membrane spectrum, which displayed a lamellar form factor characteristic of a lipid bilayer (Fig 3b), with the expected *q*^−2^ dependence at low-*q* (dashed line). A fit of the data (solid red line) revealed the average membrane hydrophobic thickness (2*D*_*C*_) to be 24.3 ± 0.9 Å at 25°C (S8 Fig). The hydrophobic thickness of the living *B*. *subtilis* membrane is thus comparable to that of synthetic phosphatidylcholine (PC) bilayers, such as dimyristoyl PC (2*D*_*C*_ = 25.7 Å at 30°C) or 1-palmitoyl-2-oleoyl PC (2*D*_*C*_ = 28.8 Å at 30°C).[46] It is also comparable to that of purified basolateral plasma membranes from rat hepatocytes (approximately 26 Å), as measured by small-angle X-ray scattering,[6] highlighting the conservation of membrane structure across animal and eubacterial kingdoms.

### Detecting lateral membrane structure in vivo using SANS

Finally, we sought to examine the lateral structure within the membrane to determine whether the lipids in the *B*. *subtilis* membrane are uniformly mixed or display nanoscopic organization, as predicted by the lipid raft hypothesis.[21] Canonical mammalian lipid domains are believed to be enriched in the high-melting lipids, cholesterol and sphingomyelin. Bacteria generally lack these lipids, but are nonetheless believed to have lipid domains[47–53] formed from lipid species playing analogous roles. [54,55] The expected hallmark of lipid domains, in any system, is the lateral separation of higher- and lower-melting lipids with associated proteins into distinct phases.

We recently introduced a contrast-variation strategy to detect lateral lipid organization in synthetic vesicles using SANS[28,29]. This strategy relies on differential H/D labeling of the lipid phases to control contrast in the plane of the membrane. With a suitable labeling strategy, the separated phases can be either contrasted or matched with respect to each other and the buffer. A particularly important case is where a mixture of lipids has an average contrast matching that of the buffer, but partitions into H- and D-enriched phases that contrast each other and the buffer. Experimentally, uniform mixing creates a null-scattering condition, whereas phase separation in the plane of the membrane (domain formation) induces neutron contrast and an observable signal in the SANS spectrum. In S9 Fig and Table F in S1 Text we present a series of similar SANS experiments which show how neutrons are sensitive to contrast in the plane of the bilayer and how neutron contrast can be controlled by thermally induced mixing of the 2 phases or by selecting specific isotopic mixtures which result in contrast-matched phases.

**Fig 4:**
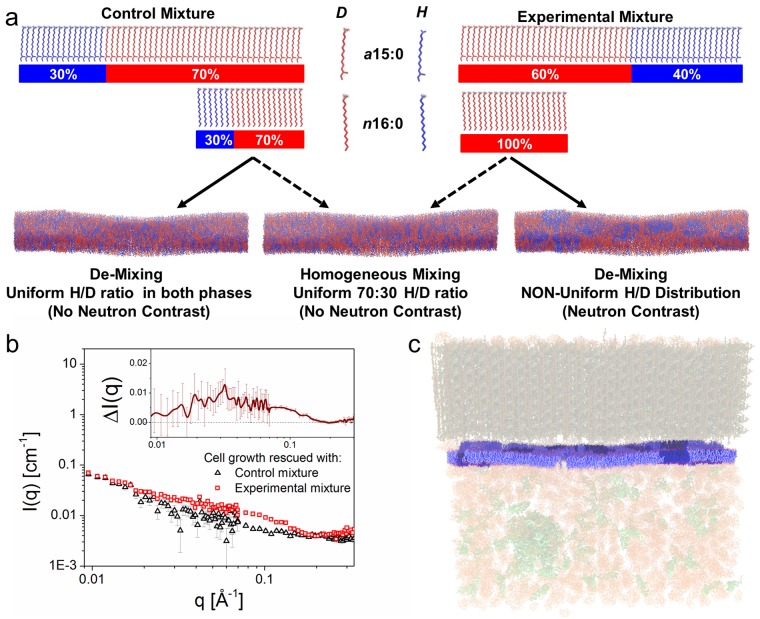
Detecting lateral lipid organization in *B*. *subtilis*. (a) Schematic of the experiment. Neutron contrast in the membrane was controlled using different blends of hydrogen (H)- and deuterium (D)-fatty acids (FAs). The control mixture consisted of anteiso-pentadecanoic acid (a15:0) and normal-hexadecanoic acid (n16:0), each 30% H and 70% D (inset to Fig 3b, red curve). This mixture is contrast-matched to 85% D_2_O and produces only background scattering whether or not the lipids are uniformly distributed (lower left and lower center panels). The experimental mixture contained the same FAs, but with a different isotopic distribution, i.e., n16:0 being 100% D and a15:0 being 40/60 H/D. In *B*. *subtilis*, this mixture produces an overall ratio of 30/70 H/D because a15:0 is more abundant (Fig 2c, bottom). If the lipids are uniformly distributed the membrane will be contrast-matched and produces no net scattering above background (indistinguishable from the control, lower center panel). However, if the lipids are organized in a manner that the high-melting n16:0 and low-melting a15:0 in membrane lipids are nonuniformly distributed, neutron contrast and a scattering signal will arise—illustrated in the lower right panel of (a) and in (c), which have patches enriched in n16:0. Just as these patches create contrast visible to the eye in the illustration, they create contrast visible to neutrons in the experiment in the form of excess scattering. (b) Small-angle neutron scattering (SANS) spectra of cerulenin-treated *B*. *subtilis* Δ*yusL*, incorporating experimental and control mixtures. The experimental mixture displays clear excess scattering (inset) that can result only from the nonuniform distribution of lipids with different contrast. From the relation ℓ = 2π/*q*, it can be inferred that the excess scattering in the range *q* = 0.01–0.15 Å^−1^ corresponds to structures with length scales of 3–40 nm. Error bars correspond to ± *σ*. See S4 Data. Gas chromatography/mass spectrometry (GC/MS) analyses of membrane FAs are provided in S10 Fig. (c) Representation of the membrane having visible lipid patches (of arbitrary structure), with the buffer, cell wall, and cytoplasmic contents all being contrast-matched.

The in vivo adaptation of this strategy compares scattering from cells with 2 different H- and D-FA mixtures that are, on average, contrast-matched to the medium (Fig 4a). In the control mixture, there can be no contrast regardless of whether or not the membrane lipids are uniformly mixed, because all species are present at the same H/D ratio. However, in the experimental mixture, de-mixing among lipids creates contrast as described above, producing a measurable increase in the scattered intensity as a result of local inhomogeneities in the H/D distribution.

We implemented this strategy in cerulenin-treated *B*. *subtilis* Δ*yusL* cells using an experimental mixture of 100% D n16:0 (high-melting) and 40/60 H/D a15:0 (low-melting). When corrected for the relative abundances of these FAs in the cell (S10 Fig), this mixture creates an average membrane contrast matching the 30/70 H/D FA ratio (a15:0 and n16:0, each 30% H and 70% D) used for the control mixture (inset to Fig 4b) as well as the rest of the cellular components, including the 85% D_2_O buffer. SANS spectra from *B*. *subtilis* fed the experimental mixture reproducibly displayed an excess scattering over the *q* range 0.015–0.2 Å^−1^ compared to cells fed the control mixture (results from a repeat experiment are shown in S11 Fig). As the only source of contrast in the experiment was the membrane H/D FA pool, this result supports the notion that there is lateral de-mixing of lipids containing the high- and low-melting FAs on a length scale ℓ of 3–40 nm (ℓ = 2π/*q*) (Fig 4c), consistent with the lipid raft hypothesis.

## Discussion

Understanding how the nanoscale structure of biological systems relates to function is a challenging, ongoing pursuit. One difficulty is that only a few probes are capable of directly interrogating structure at this scale: electrons, X-rays, and neutrons. Electrons have enjoyed the widest application in cell biology, and EM remains the single most powerful tool for studying cellular ultrastructure. With regard to the *B*. *subtilis* membrane, a cryo-EM study of sectioned, freeze-substituted cells provided a striking picture of the cellular architecture, including the cell wall.[56] However, the structure of the plasma membrane was not well determined, and its thickness was estimated at 66 ± 8 Å, a surprisingly large value. Our results complement this EM picture of the cell envelope by providing a high-resolution hydrophobic thickness determination obtained under physiological conditions.

X-ray and neutron scattering have been widely used for studying the structure of model membranes composed of defined lipid mixtures[3–5,28,29,46] or natural lipid extracts.[6–12] X-ray scattering has also been used recently for the ex vivo study of cellular membranes,[6] but its application to intact cells is confounded by the issue of background scattering from water and biomolecules. Neutron scattering uniquely provides a solution to the background problem in the form of isotopic contrast variation. We have shown here that in vivo contrast variation through metabolic labeling can effectively suppress scattering from the complex cellular milieu, while highlighting specific features of interest, even when they arise from minor components such as lipids (approximately 1% of cellular wet mass). Furthermore, the cold neutrons used for scattering experiments are well suited for studies on living cells because of their low kinetic energy (<0.025 eV) and their nonionizing character—in contrast to high-energy X-ray and electron beams (>5,000 eV).

Prior applications of neutron scattering in vivo have relied upon external solvent contrast, only, which in some cases has been sufficient to observe Bragg scattering from repeat structures in thylakoid membranes[57,58] and mitochondria.[59] These studies have not revealed membrane structure per se, but have provided information on the arrangement of closely packed membranes. By creating internal contrast, i.e., by differentially labeling specific cellular components, we have, for the first time enabled high-resolution ultrastructural measurements on a single membrane. In this work, we demonstrated the power of a chemical-biology–based approach to create selective internal contrast, thereby enabling high-resolution measurements of the in vivo membrane thickness.

Our in vivo contrast variation approach also provides a new tool to study lateral membrane structure in living cells. Neutron-based structural methods offer distinct advantages in that they report nanoscopic lipid structure directly, without the need for models or extrinsic probes. Indications of nonuniform mixing[60,61] within the plasma membrane emerged contemporaneously with the landmark fluid–mosaic model proposed in 1972,[16] the concept of membrane domains became well established by the mid-1970s,[62] and the lipid raft hypothesis was formalized in 1997.[21] Nonetheless, the existence of lipid domains has remained controversial,[63] and because they are believed to be both transient and smaller than the diffraction limit of light (200 nm), they eluded observation by conventional microscopic techniques. Recently, however, ultra-resolution fluorescence microscopy was used to identify diffusionally restricted islands on the scale of 20 nm in the plasma membrane of rat kidney epithelial cells.[6] Our observation of lipid segregation on a comparable scale in the plasma membrane of *B*. *subtilis* is consistent with the existence of analogous lipid domains in bacteria and supports the notion that nanoscopic lipid assemblies are an integral feature of biological membranes.

The critical barrier that has prevented application of high-resolution neutron scattering techniques in vivo was lack of a means to create internal contrast. In this work, we overcame the barrier and showed that *B*. *subtilis* is an ideal in vivo model system for the application of neutron contrast variation strategies. Through specific growth conditions and select genotypes, we were able to attenuate cellular contrast globally and precisely reintroduce contrast into the membrane. With the ability to control both the chemical and isotopic properties of the membrane lipids, we were able to interrogate both transverse and lateral membrane structure. The same general approach to selective contrast can potentially be extended to other biomolecules and model organisms for applications outside the membrane arena. More immediately, the in vivo experimental platform can be used to investigate the response of the plasma membrane to a diverse range of physical, chemical, genetic, and environmental stimuli. We anticipate that this capability will therefore prove valuable in many areas, such as antibiotic development, biofuel production, membrane protein function, and understanding the interplay between the membrane, cytoskeleton and cell wall in creating a protective, adaptable, multifunctional interface.

## Materials and methods

### Materials

Deuterium oxide (99.9% D) and algal amino acids (unlabeled and uniformly D-labeled, with an isotopic purity of 98%) were obtained from Cambridge Isotope Laboratories. Palmitic acid (H-n16:0) and palmitic acid-d_31_ (D-n16:0) were obtained from Sigma–Aldrich. 12-Methyltetradecanoic acid (H-a15:0) was purchased from Sigma–Aldrich or prepared from *s*-butyl magnesium chloride (Sigma–Aldrich) and 11-bromoundecanoic acid (Sigma–Aldrich) according to the method of Baer and Carney[64] and purified by vacuum distillation. Perdeuterated D-a15:0 was prepared from H-a15:0 through 3 cycles of H/D exchange with D_2_O catalyzed by 10% Pt/C at 220°C as described by Yepuri et al.,[65] followed by chromatography on silica gel and vacuum distillation. The final product was chemically homogeneous and had an isotopic purity of 99%, as determined by analysis of its derived methyl ester by GC/MS. Cerulenin was obtained from Alfa Aesar (Tewskbury, MA) and stored in the dark at –80°C as a solid. FA-free bovine serum albumin (BSA, catalog number A8806) was obtained from Sigma–Aldrich (St. Louis, MO). All other materials were obtained from commercial suppliers and used as received.

### Cultivation conditions, sample preparation, and cell viability

*B*. *subtilis* 168 (parent strain) and a Δ*yusL* mutant (strain BKE32840) were obtained from the Bacillus Genetic Stock Center (The Ohio State University, Columbus, OH). The Δ*yusL* strain lacks enoyl-CoA hydratase/3-hydroxyacyl-CoA dehydrogenase activity and is severely deficient in its ability to degrade exogenous long-chain FAs;[36] therefore, this strain was used for all experiments where exogenous FAs were supplied in the cultivation medium. General growth and maintenance of *B*. *subtilis* was performed in either Luria–Bertani (LB) rich medium or M9 minimal medium with 2% glucose supplemented with 5 mM l-tryptophan. Solid media were prepared by the addition of 1.5% Bacto or Noble Agar (Difco) to LB or M9 medium, respectively. Erythromycin was added to 0.5 μg/mL for routine maintenance of BKE32840. Cultures were incubated at 37°C, and liquid cultures were aerated by shaking at 250 rpm. Where applicable, supplemental FAs were added to a final concentration of 8 mg/L each of a15:0 and n16:0 from 25 mg/mL stock solutions in ethanol, along with 10 g/L of FA-free BSA as a carrier to aid solubility. Cerulenin (10 mg/mL in ethanol stock solution) was prepared fresh and added immediately prior to inoculation. The required final concentration was determined empirically and varied by supplier and batch. For the work described here, cerulenin from Alfa Aesar was used at a final concentration of 50 μg/mL, which was sufficient to fully suppress endogenous FA synthesis, as judged by inhibition of growth, and rescued by exogenous FA (S5 Fig).

Partially deuterated cells were grown in M9, prepared using 90% *v/v* D_2_O and H-glucose. This medium produced approximately 60%–70% deuteration of the carbon skeletons in biosynthetic molecules (analysis described below). *B*. *subtilis* 168 and BKE32840 were adapted to growth in M9-Gluc + 5 mM l-tryptophan prepared with 90% (*v/v*) D_2_O by serial passage (1:100 inoculum) in media prepared with increasing concentrations of D_2_O (H_2_O:D_2_O 100:0, 50:50, and 10:90). Cerulenin-treated, FA-supplemented cells were grown starting from a culture of untreated, unsupplemented cells. The starter culture (OD_600_ 0.8–1.0) was diluted 1:20 in cerulenin/FA medium to an OD_600_ of ca. 0.05, incubated for growth to an OD_600_ of 0.8–1.0, then passaged by dilution (1:20) into fresh medium. FA composition was monitored at each passage by GC/MS; 5–8 passages were required to clear the native FAs and adapt the cells to the FA-dependent condition. Control cultures without supplied FA (no-growth controls) were monitored in parallel for each experiment to ensure that the cerulenin remained active during the incubation period. Optical densities were determined at 600 nm using a Synergy Mx plate reader (BioTek, Winooski, VT, USA), using a 96-well microtiter plate and 300-μL well volumes.

For contrast experiments (Figs 1 and 2, and S2 Fig), *B*. *subtilis* 168 cells were resuspended in PBS (10 mM Na_2_HPO_4_, 1.8 mM KH_2_PO_4_, 137 mM NaCl, and 2.7 mM KCl), prepared either with H_2_O or D_2_O (H-PBS or D-PBS, respectively), then mixed in appropriate proportions. For all H cells (Fig 1c), 250 mL of fresh culture (OD_600_ = 1.5) in H-M9 medium was split into 2 parts, which were processed in parallel. Cells were harvested by centrifugation at 6000 × *g* for 20 min at 4°C, then washed by centrifugation/resuspension with 3 × 10 mL of H- or D-PBS, allowing 5 min for equilibration at each step. The washed cell pellets were then resuspended in 11 mL of the same buffer to provide a cell concentration approximately 50 mg/mL wet weight, equivalent to approximately 10 mg/mL dry weight. For deuterated cells (Fig 2), the same procedure was followed except that a 125-mL culture grown in D-M9 prepared with 90% D_2_O was used, providing a final cell concentration of approximately 5 mg/mL dry weight. Cell suspensions were loaded into quartz “banjo” cells (diameter 22 mm, path length 1 mm) for study by SANS. Measurements were made at 37°C, using a single detector position, and the total scattering was analyzed as described below.

For membrane structural analyses (Figs 3 and 4, S7 and S11 Figs), FA-fed *B*. *subtilis* 168 Δ*yusL* cells were grown in M9-Gluc prepared with 90% D_2_O. Cells from a 35-mL culture were harvested at an OD_600_ of 0.5, washed with 3 × 3 mL of PBS, prepared with 85% D_2_O, and resuspended in 1 mL of the same buffer, providing a final cell concentration of approximately 5 mg/mL dry weight. Because of the long data collection times required (up to 4 h), glucose (0.1% *w/v*), MgSO_4_ (10 mM), and cerulenin (50 μg/mL) were added to the final resuspension buffer, the pH was reduced to 6.8, and the measurements were made at 25°C to prolong cell viability and minimize autolysis.[39,40] As discussed below under **cellular viability and integrity** and shown in S6 and S7 Figs, cells remained >90% viable and displayed consistent SANS spectra over a period of 4 h under these conditions.

### Extraction of cellular lipids and preparation of FAMEs

Lipids were extracted from cells and characterized for FA content by GC/MS of derived FAMEs (schematic description provided in S4 Fig). Total lipid extracts were prepared using a modification[66] of the method of Bligh and Dyer.[67] In brief, cells were pelleted by centrifugation at 6000 × *g* for 15 min, followed by 3 washes in 1% (w/v) NaCl. Samples were lyophilized in 10 mL glass test tubes with Teflon-faced screw caps, to each of which was sequentially added 0.5 mL of chloroform, 1 mL of methanol, and 0.4 mL of water, with vigorous agitation at each stage. This mixture forms a single phase and was left to stand for 18 h at room temperature with occasional agitation. After 18 h, phase separation was induced by the addition of 0.5 mL of chloroform and 0.5 mL water. The lipids were recovered from the lower chloroform phase by evaporation of the solvent in a new 10-mL glass test tube under an argon stream. FAMEs were prepared by acidic methanolysis of dried lipid extracts or intact cells.[68] Solvents, if present, were evaporated under a stream of argon prior to the addition of 1 mL of concentrated HCl/methanol (10% v/v). The test tube was then purged with argon, sealed, and heated to 85°C for 2 h. After cooling, 1 mL of water and 1 mL of hexane were added, and the contents were vortex-mixed. After phase separation, a portion (approximately 700 μL) of the upper phase was taken out for GC/MS analysis.

### Preparation and derivatization of cellular amino acids

Cellular amino acids were analyzed by GC/MS, as described by Dauner and Sauer.[69] Cells from 10 mL of culture at OD_600_ ≈ 0.7 were harvested by centrifugation, washed 3 times with water by resuspension/centrifugation, and stored frozen. For analysis, cells were resuspended in 1 mL of water in a microcentrifuge tube and lysed by sonication. After centrifugation at 14,000 × *g* for 15 min, a 500-μL portion of the supernatant was transferred to a glass vial and mixed with 1.5 mL of 6 M HCl. Standard solutions were prepared from H- or D-algal amino acid mixtures and processed in the same manner. The vials were sealed with Teflon-lined caps and heated at 110°C for 24 h to hydrolyze protein, after which the volatiles were removed by rotary evaporation. The hydrolysates were resuspended in tetrahydrofuran (100 μL) and *N*-*tert*-butyldimethylsilyl-*N*-methyltrfluoroacetamide (100 μL) and then heated to 60°C for 1 h to produce *tert*-butyldimethylsilyl amino acid derivatives suitable for GC/MS analysis. Samples were diluted with hexane 1:10 (v/v) prior to analysis.

### GC/MS

GC/MS analysis was performed using an Agilent 5890A gas chromatograph with a 5975C mass-sensitive detector operating in electron-impact mode (Agilent Technologies, Santa Clara, CA). The instrument was equipped with an HP-5ms capillary column (30 m long, 0.25 mm outside diameter, and 0.25 μm coating thickness) using helium at 1 mL/min as the carrier gas. Samples of 1 μL were introduced using splitless injection at an inlet temperature of 270°C. FAMEs were eluted using a temperature program of 2 min at 60°C; 20°C/min to 170°C; 5°C/min to 240°C; and 30°C/min to 300°C for 2 min. Derivatized amino acids were eluted with a temperature program of 2 min at 100°C; 10°C/min to 280°C for 2 min.; and 25°C/min to 325°C for 2 min. Peak assignment, integration, and mass spectral analysis were performed using the instrument's ChemStation Enhanced Data Analysis software and the NIST mass spectral database. Peaks for deuterated compounds were identified on the basis of retention times and spectral comparison with nondeuterated compounds. The extent of deuteration was assessed by determining the gain in molecular mass for parent ions of FAMEs (Table B in S1 Text) or of selected fragment ions for amino acids (Table C in S1 Text).

### SANS

SANS data were collected on Bio-SANS[70] and the Extended Q-range Small Angle Neutron Spectrometer (EQ-SANS)[71] at the at the High Flux Isotope Reactor and the Spallation Neutron Source, respectively, both located at Oak Ridge National Laboratory. Two-dimensional scattering data from both instruments were reduced using the Mantid[72] software and normalized to a porous silica standard to establish an absolute scale, and corrected for pixel sensitivity, dark current, and sample transmission. Background scattering was subtracted from the 1D intensity versus *q*, which is defined as:

q= 4πsin(θ)/λ (1)

where *λ* is the neutron wavelength and 2θ is the scattering angle relative to the incident beam. At EQ-SANS, the data were collected in 60 Hz mode with 2 instrumental configurations: 1.3-m sample-to-detector distance with 4–7 Å neutrons (*q* = 0.050–0.4 Å^−1^) and 4.0 m sample-to-detector distance with 10–13.4 Å neutrons (*q* = 0.009–0.07 Å^−1^), yielding a total *q*-range from approximately 0.009 to 0.4 Å^−1^. At BioSANS, 6-Å neutrons were used at 2 sample-to-detector distances, 2.5 m (*q* = 0.050–0.30 Å^−1^) and 15.3 m (*q* = 0.005–0.060 Å^−1^), yielding a total *q*-range from 0.005 to 0.30 Å^−1^. Collection times did not exceed 4 h, at which point cells were determined to be better than 90% viable, as shown below in **cell viability and sample integrity** (S6 and S7 Figs). The scattering data from the cells demonstrate no statistically significant change in scattered intensity after 4 h (S7 Fig).

Model lipid mixtures (S9 Fig) were produced using synthetic lipids from Avanti Polar Lipids (Alabaster, AL) and prepared as follows: lipids were dissolved in chloroform, dispersed as a film by evaporation in a 20-mL scintillation vial, and dried overnight under vacuum (>6 h). The lipid films were then rehydrated in the isotopically appropriate solvent (H_2_O, D_2_O, or a combination thereof) and subjected to 5 freeze-thaw cycles prior to extrusion through 100-nm pore-diameter track-etched polycarbonate membrane filters. The final concentration of vesicles for scattering measurements was 10 mg/mL.

Data used for the contrast experiments (Figs 1 and 2) were collected on EQ-SANS for 0.009 Å^−1^ < *q* < 0.06 Å^−1^. The data were evaluated as the Porod invariant:

$$Q^{*}=\int q^{2}I\left(q\right)dq=\frac{2\pi I\left(0\right)}{V_{p}}$$ (2)

The proportionality of Q* to the total scattering intensity *I*(0) makes it a useful metric for comparison to structure-independent estimates of *I*(0) based only on composition and scattering length density, *ρ*. *V*_*p*_ is the Porod volume. To analyze the acyl thickness of the bilayer, the data were modeled using the expression:[73]

$$I\left(q\right)=\frac{N}{V}V_{s}^{2}{\left(\rho_{m}-\rho_{s}\right)}^{2}{\langle |F\left(q\right)|}^{2}\rangle$$ (3)

for an arbitrary number of bilayers, *N*, of volume, *V*_*s*_, with scattering length density *ρ*_*s*_, in a solvent of *ρ*_*m*_, where *F*(*q*) is the form factor describing the lamellar shape, and (*ρ*_*m*_ *− ρ*_*s*_), is the contrast term. The scattering law used to model the data was the lamellar form factor representing the hydrocarbon core of the bilayer, with 2 identical head group regions on either side, with scattering densities matching that of the surrounding cellular environment. Fitting of the SANS data was performed in SASview.[74]

### Estimation of molecular and cellular scattering

Estimates of total scattering from *B*. *subtilis* (Figs 1 and 2) were made as follows. A neutron scattering signal arises when there is a difference in neutron scattering length density, *ρ*, between a species, *s*, and the medium in which it resides in, *m*. The difference, (*ρ*_*m*_*−ρ*_*s*_), is called contrast, and the scattered intensity is proportional to its square. For any species,

$$\rho =(\Sigma_{i}^{n}b_{i})/V$$ (4)

where *n* is the number of atoms, *b* is the coherent neutron scattering length for each atom, and *V* is the volume of the species. Because each class of biomolecule (i.e., protein, lipid, carbohydrate, RNA, or DNA) has a nearly constant chemical composition and density, ρ is well approximated as a single value for each class (Table A in S1 Text).[30]

Deuterium labeling increases the ρ of an unlabeled molecule (*ρ*_H_) because the scattering length *b* is greater for D than H (6.67 versus –3.74 fm).[31] In biomolecules, the hydrogen atoms can be assigned to 2 categories, those bound to carbon (CH), which do not exchange with water, and those bound to heteroatoms (XH, X = N, O, or S), which do exchange with water. The fractions in the 2 categories are consistent within each class of biomolecule, which allows for the definition of the terms, Δ*ρ*_CD_ and Δ*ρ*_XD_, that represent the increase in *ρ* resulting from the complete deuteration of each category. For a deuterated biomolecule:

$$\rho =\rho_{H}+f_{XD}\Delta \rho_{XD}+f_{CD}\Delta \rho_{CD}$$ (5)

where *ρ*_H_ is *ρ* for the unlabeled (all-H) molecule, and *f*_XD_ and *f*_CD_ are the fractions of deuterium substitution in the XH and CH categories, respectively. Estimates (Figs 1b and 2a) were generated using Eq 5 and the values for *ρ*_H_, Δ*ρ*_XD_, and Δ*ρ*_CD_ in Table A in S1 Text. The differing slopes for each class of biomolecule reflect differences in Δ_XD_.

The total scattering from a cell, *I*_cell_(0), is the sum of the scattering contributions from all of the cell’s biomolecular species, given by the relation

$$I_{cell}\left(0\right)\propto \Sigma_{s}\chi_{s}{\left(\rho_{m}-\rho_{s}\right)}^{2}$$ (6)

where χ_s_ is the volume fraction of each species *s*, *ρ*_*s*_ is its neutron scattering length density, and *ρ*_*m*_ is the average neutron scattering length density of the medium (all species *s*, including intracellular water). When *ρ*_s_ = *ρ*_m_, the species is contrast-matched and thus effectively invisible to neutrons, provided the medium is uniform. The scattering attributable to an individual species *j* is given by:

$$I_{j}\left(0\right)\propto 0.5\chi_{j}\Sigma_{s}\chi_{s}{\left(\rho_{j}-\rho_{s}\right)}^{2}$$ (7)

In an undeuterated cell, the χ_*j*_ for biomolecules is sufficiently high and the *ρ*_*j*_ is sufficiently different that the surrounding medium is not truly uniform. As a result, the total scattering for a given species *j* reflects interspecies contrast and is not completely nulled at the nominal contrast matchpoint defined by the average of the medium (χ_j_ = χ_m_). However, judicious deuteration can be used to converge ρ for water and most biomolecules (cf. Figs 1b and 2a), effectively suppressing the interspecies contributions.

Knowledge of the cell’s composition (Table A in S1 Text) allows one to estimate the total cellular scattering—broken down by biomolecular species as a function of deuteration in the solvent and in the CH skeletons of the various biomolecules (Figs 1c and 2d). Approximately 80% of the dry mass of a *B*. *subtilis* cell is made up of protein (53%), RNA (18%), DNA (2.6%), lipid (5.2%), and carbohydrate (2.8%).[33] The remaining mass—which was neglected in our estimates—consists of small organic molecules, such as amino acids, cofactors, and nucleotides plus inorganic material. These data were used with Eqs 6 and 7 to calculate the predicted total scattering in Fig 1c.

Estimated scattering for cells grown in D-M9 (Fig 2b) relied on analyses of lipids and proteins extracted from *B*. *subtilis* under relevant conditions to adjust *ρ* according to Eq 5. The net deuteration of the FA pool in the absence of cerulenin and supplemental FAs was 68.5%, with deuteration of individual FAs ranging from 66% to 71% (Table B in S1 Text). The deuteration of the amino acid pool was more variable and had a lower average deuteration of 60% (Table C in S1 Text), which is similar to what would be expected in *Escherichia coli*.[75] A value of 70% was assumed for other biomolecules, and the extent of deuteration at water-exchangeable positions was assumed to match that of the medium.

### Cell viability and sample integrity

Cell viability was evaluated using optical density measurements, manual cell counts with a hemocytometer, and fluorescent live/dead staining with the BacLight Bacterial Viability Kit (Catalog number L7012, Molecular Probes, Eugene, OR). The live/dead assay uses a mixture of 2 fluorescent nucleic acid stains (SYTO 9 and propidium iodide), which stain live cells green and dead cells with compromised membranes, red. Fluorescence micrographs of stained cells were acquired with a Zeiss Axioskop 2 Plus and analyzed with ImageJ[76] to count cells using green and red fluorescence channels. Nonirradiated suspensions of the Δ*yusL* strain prepared identically to the samples used for neutron beam experiments were used as controls. Cell suspensions were incubated in a sealed cuvette at 25°C, and optical density (OD_600_) was recorded at 1-min intervals over a period of 24 h (S6a Fig). Direct cell counts and live/dead staining of the nonirradiated samples were performed immediately after processing and at 4 h and 24 h (S6b Fig). These experiments showed close correspondence among the 3 measures of viability and that cells remained 90% viable over 4 h and 50%–60% viable over the course of 24 h under the conditions of the experiment. Irradiated cell suspensions were taken from the neutron beam after data collection and allowed to decay for approximately 30 min, at which time they were subjected to a radiological survey. Radiological safety protocols do not permit timely removal of cell suspensions for microscopic examination. Instead, optical density measurements of the cells at 4 h and 24 h were carried out using a Shimadzu UV-2700 UV–Vis spectrophotometer (S6a Fig). FA stability was monitored over the course of the 4 h it took to collect a complete SANS data set using GC/MS as described above for nonirradiated samples (S7 Fig). Finally, cell stability in the neutron beam was assessed by repeating the SANS measurement 3.5 h after the cells were put in the beam showing no change in the scattered intensity (S7 Fig).

## Supporting information

S1 Fig Relative sensitivity of different probes to elemental composition. For neutrons and X-rays, radii of the circles are scaled to scattering lengths, which are proportional to atomic number Z for X-rays, and unrelated to *Z* for neutrons. Hydrogen (^1^H) is distinct from the other elements shown in that its scattering length *b* is negative, and the corresponding circle is colored red to note this distinction. For electrons, areas are scaled to elastic scattering cross-sections, which are approximately proportional to *Z*^3/2^.[77] Scattering power is normalized to oxygen (*Z* = 8).
(TIF)

S2 Fig Raw SANS data. Raw data for contrast series experiments are shown in the main text as (**a**) **Figs. 1c and** (**b and c**)**2d**. Small angle scattering was measured as a function of % D_2_O in buffer. Data in S5 Data, to be corrected for the instrumental and solvent backgrounds, and scaled to a porous silica standard. The Porod invariant was evaluated from these measurements as in the observed *q*-range from 0.009 Å^−1^ < *q* < 0.06 Å^−1^. This quantity, Q*, is directly proportional to *I*(0) [*I*(0)/Q* = *V*_P_/2π^2^, where *V*_P_ is the Porod volume), making it a useful metric for comparison to estimated values of *I*(0).
(TIF)

S3 Fig Schematic of fatty acid extraction and methanolysis protocol. The total lipid fraction was extracted from an aliquot of cells using the Bligh-Dyer method. Following extraction, the lower lipid containing phase was dried under argon, and the lipids were heated in acidic methanol to create fatty acid methyl esters, which were then extracted using hexane and analyzed using GC/MS.
(TIF)

S4 Fig Summary of GC/MS analysis of lipid extracts of unmodified *B*. *subtilis* grown in H_2_O and 90% D_2_O conditions. (a) GC/MS total ion chromatograms for FAMEs extracted from *B*. *subtilis* grown in M9 medium prepared with H-glucose and either H_2_O (black, top) or 90% D_2_O (red, bottom). The phospholipids in the native membrane of *B*. *subtilis* contain a mixture of saturated linear (normal) and branched chain (iso- or anteiso-) fatty acids shown in (b). As expected, 7 FAMEs were observed from cells cultured in 90% D_2_O (a, lower panel). Deuterated FAMEs eluted earlier, and their associated peaks were broader due to the presence of multiple isotopomers for each species. (c) and (d) show mass spectra for each FAME from cells grown in H- or D-medium, respectively. From the spectra, the extent of deuteration was determined by noting the change in mass of the molecular ion [M]^+•^ (Table B in S1 Text and S2 Data). The distribution of isotopmers is shown in (d).
(TIF)

S5 Fig FA-dependent growth of *B*. *subtilis* BKE32840 in the presence of cerulenin. Cell growth was strongly inhibited in the presence of cerulenin (Cer; 50 μg/mL). However, growth could be rescued by the addition of exogenous fatty acids (*a*15:0 + *n*16:0) in the presence of fatty acid-free BSA as a carrier, establishing chemical and isotopic control of the cell membrane FA composition. Shown are the end-point optical densities (OD_600_) for cells grown in M9 minimal medium containing 5 mM l-tryptophan, 2% glucose (except where indicated), and the indicated additives (see S6 Data). End-point measurements were recorded at 24 h for control conditions (BSA only), and at 64 h for the other conditions–under which the cells grew more slowly. All experiments were carried out in triplicate.
(TIF)

S6 Fig Stability and viability of *B*. *subtilis* cells in suspension. For SANS studies at 25°C, cells were suspended in 85% D_2_O PBS (pH 6.8) containing glucose (0.1% w/v), MgSO_4_ (10 mM), and cerulenin (50 μg/mL), and were then transferred to banjo-shaped quartz cuvettes with 1 or 2 mm beam paths. Once the cells were transferred, the cuvettes were sealed. SANS measurements were made over a maximum 4 h period. (a) OD_600_ drops off slowly over the first 8 h, and more rapidly thereafter. The blue diamonds denote OD_600_ measurements taken of an irradiated sample whose SANS spectrum is shown in Fig 4. The drop in OD_600_ over the 4 h measurement period was 5% for the sample in the beam and 7% for the control (non-irradiated), continuously monitored sample. (b) Cell densities were also determined through direct counts made using a hemocytometer on aliquots of the control sample from (a). Cell densities are consistent with OD_600_measurements. (c) The fraction of intact cells (which also happen to be alive) was quantified using a standard live/dead stain on aliquots of the continuously monitored sample from (a). Over the 24 h period of observation, >90% of intact cells stained green, indicating excellent cell viability and membrane integrity. After 4 h, which corresponds to maximum time that the cells were exposed to neutrons, 95% of the cells were alive (see S6 Data). (d-f) Representative false-colored, superimposed, red- and green-channel fluorescence micrographs corresponding to results shown in (c). Live cells appear green and dead cells appear red, the scale bars represents 20 μm.
(TIF)

S7 Fig Sample stability: FA content and repeat scattering measurements. Fatty acid content over the period of the measurements was assessed by extracting the membrane lipids, performing an acidic methanolysis, and quantifying the FAME content using GC/MS. (a) Total ion chromatograms are shown for the lipids extracted from *B*. *subtilis* at harvest, after 4 hours of incubation–i.e., conditions which paralleled those of the SANS measurements–and after 24 h of incubation in modified PBS buffer. (b) Integrated peak areas for the chromatograms in (a). Less than 1% change is observed for any FA after 4 h, and only a 1–2% change after 24 h out of culture. (c) A repeat scattering measurement (pink) made 2 h after the initial measurement is superimposed onto the data shown in Fig 3 of the main text (blue). This scattering result shows that the sample is stable over the course of the 4 hour data collection period (see S6 Data). Note: the statistics of the repeat measurement are poorer (hence noise is greater), simply due to the shorter collection time.
(TIF)

S8 Fig Data treatment to obtain the scattering of the *B*. *subtilis* cell membrane. (**a**) The residual background was recorded using cerulenin-treated Δ*yusL* cells, which were fed a mixture of FAs contrast-matched to 85% D_2_O (*a*15:0 and *n*16:0, each 30% H and 70% D), sample scattering was recorded from cells cultured identically, except for being provided H-*a*15:0 and H-*n*16:0 in the culture medium. Subtraction of the background from the sample scattering revealed a pure membrane spectrum, which displayed a lamellar form factor characteristic of a lipid bilayer (Fig 3b). (**b**) Fitting of the lamellar form factor with fits illustrating the model envelope ± 2σ and ± 4σ superimposed on the data.
(TIF)

S9 Fig Contrast variation strategy used to detect lateral lipid organization. We demonstrate how neutron contrast can be manipulated by isotopic substitution to reveal lateral de-mixing using vesicles of POPC/DSPC/Chol. (see Table F in S1 Text and S7 Data). Detection of de-mixing requires observations of the bilayer in two states, i.e., mixed (pink) and de-mixed (red and blue). This can be accomplished using two strategies. The first (a, upper row) compares a system in two different states (i.e., mixed and de-mixed). At high temperature, the different NSLD lipids are uniformly mixed, with their average NSLD matching that of the buffer. Lowering the temperature causes the lipids to de-mix. Although the average lipid NSLD has not changed, the domains and surround have NSLDs which differ from the buffer’s NSLD and each other, resulting in excess scattering. We performed this experiment, and the results are shown in (b). Although this approach can be used successfully when temperature is a variable, it cannot be applied to live cells, as temperature changes can adversely affect their viability. Alternatively (a, lower row), one can construct a system where the domains and surround have the same average NSLD, which matches the buffer. This is achieved by providing all components at the same isotopic ratio, so that the same NSLD is kept whether or not the lipids are mixed or de-mixed. In this scenario, the scattering signal is the same for both states, as shown in (c). This strategy was adapted for the *B*. *subtilis* experiments at 25°C, where only isotopic substitution was used to manipulate contrast in the bilayer.
(TIF)

S10 Fig GC/MS analysis of FAMEs from *B*. *subtilis*. An aliquot from samples used in Fig 4 (main text) was analyzed for FA content. The GC/MS data show that the expected ratios of H- and D-FAs were incorporated in the cell’s membrane (see S8 Data). Peak areas are listed in Table E in S1 Text.
(TIF)

S11 Fig Repeat SANS observation of lateral de-mixing of fatty acids in *B*. *subtilis*. (**a**) The experiments shown in Fig 4 were repeated. The same excess scattering is observed in the *q*-range of ~0.015 to 0.15 Å^−1^, confirming the presence of nanoscopic features on the order of 40 nm. (**b**) GC/MS chromatograms for the lipids in these membranes (colors correspond to the spectra in panel (**a**), showing the expected H/D distribution of fatty acids, analogous to that shown in S10 Fig (see S8 Data).
(TIF)

S1 Text Supporting data tables. (DOCX)

S1 Data Supplementary data 1. (XLSX)

S2 Data Supplementary data 2. (XLSX)

S3 Data Supplementary data 3. (XLSX)

S4 Data Supplementary data 4. (XLSX)

S5 Data Supplementary data 5. (XLSX)

S6 Data Supplementary data 6. (XLSX)

S7 Data Supplementary data 7. (XLSX)

S8 Data Supplementary data 8. (XLSX)

S1 Movie Summary movie. (MP4)

## Abbreviations

Å: Ångstroms
a15:0: anteiso-pentadecanoic acid
D: deuterium
D-M9: deuterium-enriched M9 minimal medium
EM: electron microscopy
FA: fatty acid
FAME: fatty acid methyl ester
GC/MS: gas chromatography/mass spectrometry
H: hydrogen
H-M9: standard M9 minimal medium
n16:0: normal-hexadecanoic acid
PBS: phosphate buffered saline
PC: phosphatidylcholine
SANS: small-angle neutron scattering
*ρ*: neutron scattering length density

## References

[1] Henderson R, Unwin PNT. Three-Dimensional Model of Purple Membrane Obtained by Electron Microscopy. Nature. 1975;257(5521):28–32. 1161000 10.1038/257028a0

[2] Edidin M. The State of Lipid Rafts: From Model Membranes to Cells. Annu Rev Biophys Biomol Struct. 2003;32:257–83. 10.1146/annurev.biophys.32.110601.142439 12543707

[3] Nagle JF, Tristram-Nagle S. Structure of Lipid Bilayers. Biochim Biophys Acta, Rev Biomembr. 2000;1469(3):159–95. 10.1016/s0304-4157(00)00016-2 PMC2747654 11063882

[4] Petrache HI, Dodd SW, Brown MF. Area Per Lipid and Acyl Length Distributions in Fluid Phosphatidylcholines Determined by ^2^H NMR Spectroscopy. Biophys J. 2000;79(6):3172–92. 10.1016/S0006-3495(00)76551-9 11106622 PMC1301193

[5] Seelig J, Seelig A. Lipid Conformation in Model Membranes and Biological Membranes. Q Rev Biophys. 1980;13(1):19–61. 7220788 10.1017/s0033583500000305

[6] Mitra K, Ubarretxena-Belandia I, Taguchi T, Warren G, Engelman DM. Modulation of the Bilayer Thickness of Exocytic Pathway Membranes by Membrane Proteins Rather Than Cholesterol. Proc Natl Acad Sci U S A. 2004;101(12):4083–8. 10.1073/pnas.0307332101 15016920 PMC384699

[7] Zaccai G, Blasie J, Schoenborn B. Neutron Diffraction Studies on the Location of Water in Lecithin Bilayer Model Membranes. Proc Natl Acad Sci U S A. 1975;72(1):376–80. 16592215 10.1073/pnas.72.1.376 PMC432308

[8] Herbette L, DeFoor P, Fleischer S, Pascolini D, Scarpa A, Blasie J. The Separate Profile Structures of the Functional Calcium Pump Protein and the Phospholipid Bilayer within Isolated Sarcoplasmic Reticulum Membranes Determined by X-Ray and Neutron Diffraction. Biochim Biophys Acta—Biomembr. 1985;817(1):103–22. 10.1016/0005-2736(85)90073-2 3159429

[9] Wilkins M, Blaurock A, Engelman D. Bilayer Structure in Membranes. Nature. 1971;230(11):72–6. 10.1038/newbio230072a0 5279041

[10] Schwartz S, Cain J, Dratz E, Blasie J. An Analysis of Lamellar X-Ray Diffraction from Disordered Membrane Multilayers with Application to Data from Retinal Rod Outer Segments. Biophys J. 1975;15(12):1201–33. 10.1016/S0006-3495(75)85895-4 1203447 PMC1334803

[11] Engelman DM. X-Ray Diffraction Studies of Phase Transitions in the Membrane of *Mycoplasma laidlawii*. J Mol Biol. 1970;47(1):115IN13117–116. 5413340 10.1016/0022-2836(70)90407-9

[12] Engelman DM. Lipid Bilayer Structure in the Membrane of *Mycoplasma laidlawii*. J Mol Biol. 1971;58(1):153–65. 5088924 10.1016/0022-2836(71)90238-5

[13] Eggeling C, Ringemann C, Medda R, Schwarzmann G, Sandhoff K, Polyakova S, et al Direct Observation of the Nanoscale Dynamics of Membrane Lipids in a Living Cell. Nature. 2009;457(7233):1159–62. 10.1038/nature07596 19098897

[14] Brown DA, London E. Functions of Lipid Rafts in Biological Membranes. Annu Rev Cell Dev Biol. 1998;14:111–36. 10.1146/annurev.cellbio.14.1.111 9891780

[15] Op den Kamp JAF. Lipid Asymmetry in Membranes. Annu Rev Biochem. 1979;48:47–71. 10.1146/annurev.bi.48.070179.000403 382989

[16] Singer S, Nicolson GL. The Fluid Mosaic Model of the Structure of Cell Membranes. Science. 1972;175:720–31. 4333397 10.1126/science.175.4023.720

[17] Spector AA, Yorek MA. Membrane Lipid Composition and Cellular Function. J Lipid Res. 1985;26(9):1015–35. 3906008

[18] van Meer G, Voelker DR, Feigenson GW. Membrane Lipids: Where They Are and How They Behave. Nat Rev Mol Cell Biol. 2008;9(2):112–24. 10.1038/nrm2330 18216768 PMC2642958

[19] Nickels JD, Smith JC, Cheng X. Lateral Organization, Bilayer Asymmetry, and Inter-Leaflet Coupling of Biological Membranes. Chem Phys Lipids. 2015;192:87–99. 10.1016/j.chemphyslip.2015.07.012 26232661

[20] Jacobson K, Mouritsen OG, Anderson RGW. Lipid Rafts: At a Crossroad between Cell Biology and Physics. Nat Cell Biol. 2007;9(1):7–14. 10.1038/ncb0107-7 17199125

[21] Simons K, Ikonen E. Functional Rafts in Cell Membranes. Nature. 1997;387(6633):569–72. 10.1038/42408 9177342

[22] Allen JA, Halverson-Tamboli RA, Rasenick MM. Lipid Raft Microdomains and Neurotransmitter Signalling. Nat Rev Neurosci. 2007;8(2):128–40. 10.1038/nrn2059 17195035

[23] Shaw AS. Lipid Rafts: Now You See Them, Now You Don't. Nat Immunol. 2006;7(11):1139–42. 10.1038/ni1405 17053798

[24] Simons K, Ehehalt R. Cholesterol, Lipid Rafts, and Disease. J Clin Invest. 2002;110(5):597–603. 10.1172/JCI16390 12208858 PMC151114

[25] Simons K, Toomre D. Lipid Rafts and Signal Transduction. Nat Rev Mol Cell Biol. 2000;1(1):31–9. 10.1038/35036052 11413487

[26] Lingwood D, Simons K. Lipid Rafts as a Membrane-Organizing Principle. Science. 2010;327(5961):46–50. 10.1126/science.1174621 20044567

[27] Mukherjee S, Maxfield FR. Membrane Domains. Annu Rev Cell Dev Biol. 2004;20:839–66. 10.1146/annurev.cellbio.20.010403.095451 15473862

[28] Heberle F, Petruzielo R, Pan J, Drazba P, Kučerka N, Standaert R, et al Bilayer Thickness Mismatch Controls Domain Size in Model Membranes. J Am Chem Soc. 2013;135(18):6853–9. 10.1021/ja3113615 23391155

[29] Nickels JD, Cheng X, Mostofian B, Stanley C, Lindner B, Heberle FA, et al Mechanical Properties of Nanoscopic Lipid Domains. J Am Chem Soc. 2015;137(50):15772–80. 10.1021/jacs.5b08894 26415030

[30] Jacrot B. The Study of Biological Structures by Neutron Scattering from Solution. Rep Prog Phys. 1976;39(10):911–53.

[31] Sears VF. Neutron Scattering Lengths and Cross Sections. Neutron News. 1992;3(3):26–37.

[32] Marty V, Jasnin M, Fabiani E, Vauclare P, Gabel F, Trapp M, et al Neutron Scattering: A Tool to Detect in vivo Thermal Stress Effects at the Molecular Dynamics Level in Micro-Organisms. J R Soc, Interface. 2013;10(82):20130003. 23446053 10.1098/rsif.2013.0003 PMC3627083

[33] Bishop D, Rutberg L, Samuelsson B. The Chemical Composition of the Cytoplasmic Membrane of *Bacillus subtilis*. Eur J Biochem. 1967;2(4):448–53. 4295208 10.1111/j.1432-1033.1967.tb00158.x

[34] Kaneda T. Biosythesis of Branched Chain Fatty Acids. I. Isolation and Identification of Fatty Acids from *Bacillus subtilis* (ATCC 7059). J Biol Chem. 1963;238(4):1222–8. 13962195

[35] Op den Kamp JAF, Redai I, van Deenen LLM. Phospholipid Composition of *Bacillus subtilis*. J Bacteriol. 1969;99(1):298–303. 4979443 10.1128/jb.99.1.298-303.1969 PMC250003

[36] Matsuoka H, Hirooka K, Fujita Y. Organization and Function of the Ysia Regulon of *Bacillus subtilis* Involved in Fatty Acid Degradation. J Biol Chem. 2007;282(8):5180–94. 10.1074/jbc.M606831200 17189250

[37] Price AC, Choi K-H, Heath RJ, Li Z, White SW, Rock CO. Inhibition of β-Ketoacyl-Acyl Carrier Protein Synthases by Thiolactomycin and Cerulenin Structure and Mechanism. J Biol Chem. 2001;276(9):6551–9. 10.1074/jbc.M007101200 11050088

[38] Wille W, Eisenstadt E, Willecke K. Inhibition of De Novo Fatty Acid Synthesis by the Antibiotic Cerulenin in *Bacillus subtilis*: Effects on Citrate-Mg^2+^ Transport and Synthesis of Macromolecules. Antimicrob Agents Chemother. 1975;8(3):231–7. 810081 10.1128/aac.8.3.231 PMC429299

[39] Jolliffe LK, Doyle RJ, Streips UN. The Energized Membrane and Cellular Autolysis in *Bacillus subtilis*. Cell. 1981;25(3):753–63. 6793239 10.1016/0092-8674(81)90183-5

[40] Svarachorn A, Shinmyo A, Tsuchido T, Takano M. Autolysis of *Bacillus subtilis* Induced by Monovalent Cations. Appl Microbiol Biotechnol. 1989;30(3):299–304.

[41] Balgavý P, Dubničková M, Kučerka N, Kiselev MA, Yaradaikin SP, Uhríková D. Bilayer Thickness and Lipid Interface Area in Unilamellar Extruded 1, 2-Diacylphosphatidylcholine Liposomes: A Small-Angle Neutron Scattering Study. Biochim Biochim Acta—Biomembr. 2001;1512(1):40–52. 10.1016/s0005-2736(01)00298-x 11334623

[42] Kučerka N, Nagle JF, Feller SE, Balgavý P. Models to Analyze Small-Angle Neutron Scattering from Unilamellar Lipid Vesicles. Physical Review E. 2004;69(5):051903. 10.1103/PhysRevE.69.051903 15244843

[43] Marquardt D, Heberle FA, Nickels JD, Pabst G, Katsaras J. On Scattered Waves and Lipid Domains: Detecting Membrane Rafts with X-Rays and Neutrons. Soft Matter. 2015;11(47):9055–72. 10.1039/c5sm01807b 26428538 PMC4719199

[44] Kučerka N, Nagle JF, Sachs JN, Feller SE, Pencer J, Jackson A, et al Lipid Bilayer Structure Determined by the Simultaneous Analysis of Neutron and X-Ray Scattering Data. Biophys J. 2008;95(5):2356–67. 10.1529/biophysj.108.132662 18502796 PMC2517047

[45] Nallet F, Laversanne R, Roux D. Modelling X-Ray or Neutron Scattering Spectra of Lyotropic Lamellar Phases: Interplay between Form and Structure Factors. J Phys II. 1993;3(4):487–502.

[46] Kučerka N, Nieh M-P, Katsaras J. Fluid Phase Lipid Areas and Bilayer Thicknesses of Commonly Used Phosphatidylcholines as a Function of Temperature. Biochim Biophys Acta, Biomembr. 2011;1808(11):2761–71. 10.1016/j.bbamem.2011.07.022 21819968

[47] Mileykovskaya E, Dowhan W. Visualization of Phospholipid Domains in *Escherichia coli* by Using the Cardiolipin-Specific Fluorescent Dye 10-*N*-Nonyl Acridine Orange. J Bacteriol. 2000;182(4):1172–5. 10648548 10.1128/jb.182.4.1172-1175.2000 PMC94398

[48] Kawai F, Shoda M, Harashima R, Sadaie Y, Hara H, Matsumoto K. Cardiolipin Domains in *Bacillus subtilis* Marburg Membranes. J Bacteriol. 2004;186(5):1475–83. 10.1128/JB.186.5.1475-1483.2004 14973018 PMC344405

[49] Matsumoto K, Kusaka J, Nishibori A, Hara H. Lipid Domains in Bacterial Membranes. Mol Microbiol. 2006;61(5):1110–7. 10.1111/j.1365-2958.2006.05317.x 16925550

[50] Donovan C, Bramkamp M. Characterization and Subcellular Localization of a Bacterial Flotillin Homologue. Microbiology (London, U K). 2009;155:1786–99. 10.1099/mic.0.025312-0 19383680

[51] López D, Kolter R. Functional Microdomains in Bacterial Membranes. Genes Dev. 2010;24(17):1893–902. 10.1101/gad.1945010 20713508 PMC2932971

[52] Bach JN, Bramkamp M. Flotillins Functionally Organize the Bacterial Membrane. Mol Microbiol. 2013;88(6):1205–17. 10.1111/mmi.12252 23651456

[53] Bramkamp M, Lopez D. Exploring the Existence of Lipid Rafts in Bacteria. Microbiol Mol Biol Rev. 2015;79(1):81–100. 10.1128/MMBR.00036-14 25652542 PMC4342107

[54] LaRocca TJ, Crowley JT, Cusack BJ, Pathak P, Benach J, London E, et al Cholesterol Lipids of *Borrelia burgdorferi* Form Lipid Rafts and Are Required for the Bactericidal Activity of a Complement-Independent Antibody. Cell Host Microbe. 2010;8(4):331–42. 10.1016/j.chom.2010.09.001 20951967 PMC3010898

[55] Sáenz JP, Grosser D, Bradley AS, Lagny TJ, Lavrynenko O, Broda M, et al Hopanoids as Functional Analogues of Cholesterol in Bacterial Membranes. Proc Natl Acad Sci U S A. 2015;112(38):11971–6. 10.1073/pnas.1515607112 26351677 PMC4586864

[56] Matias VRF, Beveridge TJ. Cryo-Electron Microscopy Reveals Native Polymeric Cell Wall Structure in *Bacillus subtilis* 168 and the Existence of a Periplasmic Space. Mol Microbiol. 2005;56(1):240–51. 10.1111/j.1365-2958.2005.04535.x 15773993

[57] Liberton M, Page LE, O'Dell WB, O'Neill H, Mamontov E, Urban VS, et al Organization and Flexibility of Cyanobacterial Thylakoid Membranes Examined by Neutron Scattering. J Biol Chem. 2013;288(5):3632–40. 10.1074/jbc.M112.416933 23255600 PMC3561581

[58] Nagy G, Posselt D, Kovacs L, Holm JK, Szabo M, Ughy B, et al Reversible Membrane Reorganizations During Photosynthesis in Vivo: Revealed by Small-Angle Neutron Scattering. Biochem J. 2011;436:225–30. 10.1042/BJ20110180 21473741

[59] Murugova TN, Solodovnikova IM, Yurkov VI, Gordeliy VI, Kuklin AI, Ivankov OI, et al Potentials of Small-Angle Neutron Scattering for Studies of the Structure of “Live” Mitochondria. Neutron News. 2011;22(3):11–4.

[60] Stier A, Sackmann E. Spin Labels as Enzyme Substrates Heterogeneous Lipid Distribution in Liver Microsomal Membranes. Biochim Biophys Acta, Biomembr. 1973;311(3):400–8. 10.1016/0005-2736(73)90320-9 4354130

[61] Verkleij A, Zwaal R, Roelofsen B, Comfurius P, Kastelijn D, Van Deenen L. The Asymmetric Distribution of Phospholipids in the Human Red Cell Membrane. A Combined Study Using Phospholipases and Freeze-Etch Electron Microscopy. Biochim Biophys Acta, Biomembr. 1973;323(2):178–93. 10.1016/0005-2736(73)90143-0 4356540

[62] Klausner R, Kleinfeld A, Hoover R, Karnovsky MJ. Lipid Domains in Membranes. Evidence Derived from Structural Perturbations Induced by Free Fatty Acids and Lifetime Heterogeneity Analysis. J Biol Chem. 1980;255(4):1286–95. 7354027

[63] Munro S. Lipid Rafts: Elusive or Illusive? Cell. 2003;115(4):377–88. 14622593 10.1016/s0092-8674(03)00882-1

[64] Baer TA, Carney RL. Copper Catalyzed Reaction of Grignard Reagents with Chloromagnesium Salts of Ω-Bromoacids. Tetrahedron Lett. 1976;17(51):4697–700.

[65] Yepuri NR, Jamieson SA, Darwish TA, Rawal A, Hook JM, Thordarson P, et al Synthesis of Per-Deuterated Alkyl Amines for the Preparation of Deuterated Organic Pyromellitamide Gelators. Tetrahedron Lett. 2013;54(20):2538–41.

[66] Lewis T, Nichols PD, McMeekin TA. Evaluation of Extraction Methods for Recovery of Fatty Acids from Lipid-Producing Microheterotrophs. J Microbiol Methods. 2000;43(2):107–16. 11121609 10.1016/s0167-7012(00)00217-7

[67] Bligh EG, Dyer WJ. A Rapid Method of Total Lipid Extraction and Purification. Can J Biochem Physiol. 1959;37(8):911–7. 10.1139/o59-099 13671378

[68] Ichihara Ki, Fukubayashi Y. Preparation of Fatty Acid Methyl Esters for Gas-Liquid Chromatography. J Lipid Res. 2010;51(3):635–40. 10.1194/jlr.D001065 19759389 PMC2817593

[69] Dauner M, Sauer U. Gc-Ms Analysis of Amino Acids Rapidly Provides Rich Information for Isotopomer Balancing. Biotechnol Prog. 2000;16(4):642–9. 10.1021/bp000058h 10933840

[70] Lynn GW, Heller W, Urban V, Wignall GD, Weiss K, Myles DAA. Bio-Sans—a Dedicated Facility for Neutron Structural Biology at Oak Ridge National Laboratory. Phys B (Amsterdam, Neth). 2006;385:880–2.

[71] Zhao JK, Gao CY, Liu D. The Extended Q-Range Small-Angle Neutron Scattering Diffractometer at the Sns. J Appl Crystallogr. 2010;43(5 Part 1):1068–77.

[72] O'Donnell A, Nahaie M, Goodfellow M, Minnikin D, Hajek V. Numerical Analysis of Fatty Acid Profiles in the Identification of Staphylococci. Microbiology. 1985;131(8):2023–33. 10.1099/00221287-131-8-2023 4056741

[73] Guinier A, Fournet G. Small Angle Scattering of X-Rays. New York: J. Wiley & Sons; 1955.

[74] Pencer J, Mills T, Anghel V, Krueger S, Epand RM, Katsaras J. Detection of Submicron-Sized Raft-Like Domains in Membranes by Small-Angle Neutron Scattering. Eur Phys J E. 2005;18:447–58. 10.1140/epje/e2005-00046-5 16292472

[75] Leiting B, Marsilio F, O'Connell JF. Predictable Deuteration of Recombinant Proteins Expressed in *Escherichia coli*. Anal Biochem. 1998;265(2):351–5. 10.1006/abio.1998.2904 9882413

[76] Schneider CA, Rasband WS, Eliceiri KW. NIH Image to ImageJ: 25 Years of Image Analysis. Nat Methods. 2012;9(7):671–5. 22930834 10.1038/nmeth.2089 PMC5554542

[77] Langmore JP, Wall J, Isaacson MS. Collection of Scattered Electrons in Dark Field Electron Microscopy. 1. Elastic-Scattering. Optik. 1973;38(4):335–50.

