# Supplementary figures and images for: The in vivo structure of biological membranes and evidence for lipid domains
Source: PLoS Biol. 2017 May 23;15(5):e2002214. doi: 10.1371/journal.pbio.2002214 (PMC5441578; doi:10.1371/journal.pbio.2002214)

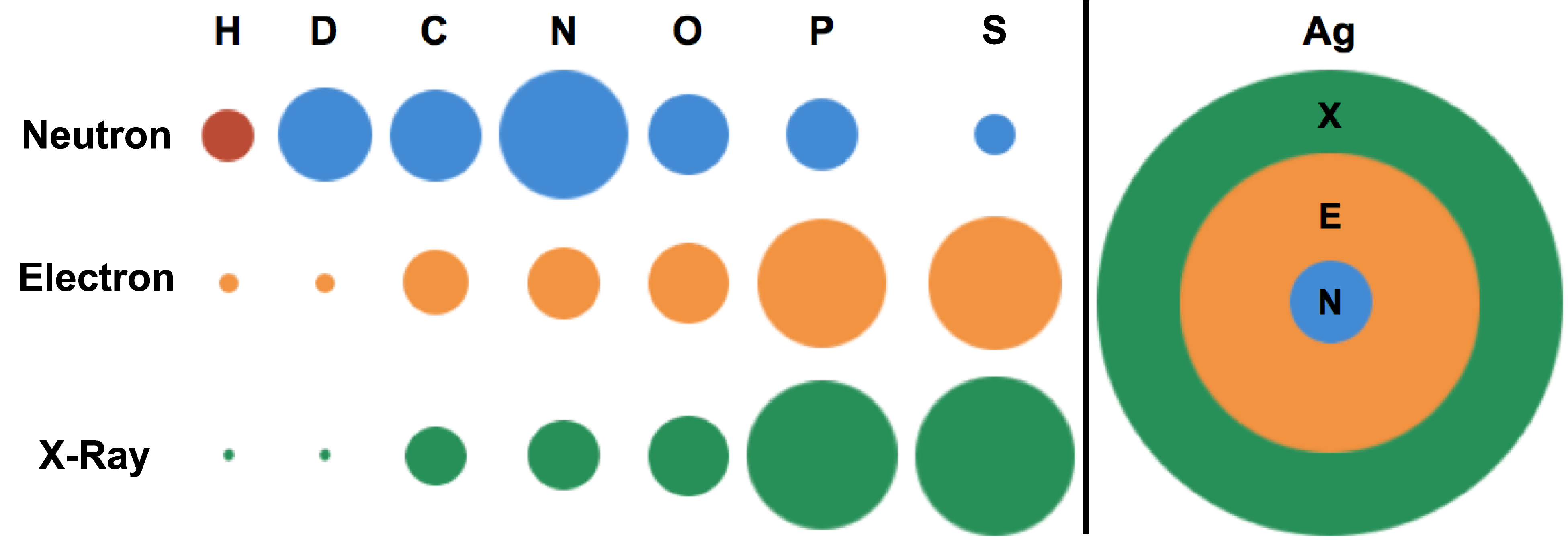

Supplement: S1 Fig — For neutrons and X-rays, radii of the circles are scaled to scattering lengths, which are proportional to atomic number Z for X-rays, and unrelated to Z for neutrons. Hydrogen (1H) is distinct from the other elements shown in that its scattering length b is negative, and the corresponding circle is colored red to note this distinction. For electrons, areas are scaled to elastic scattering cross-sections, which are approximately proportional to Z3/2.[77] Scattering power is normalized to oxygen (Z = 8). (TIF) [file pbio.2002214.s001.tif]

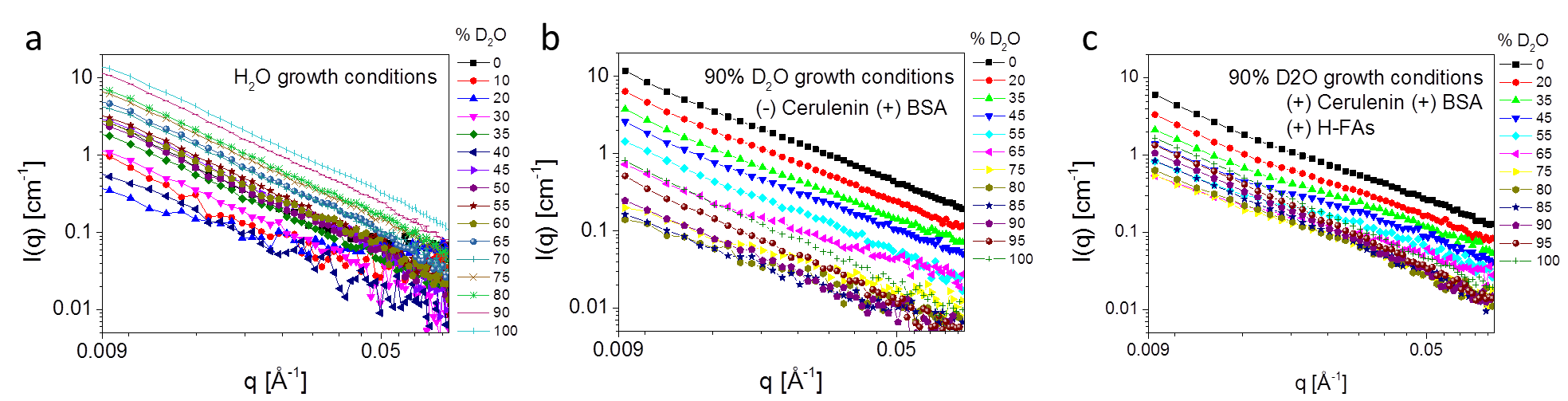

Supplement: S2 Fig — Raw data for contrast series experiments are shown in the main text as (a) Figs. 1c and (b and c)2d. Small angle scattering was measured as a function of % D2O in buffer. Data in S5 Data, to be corrected for the instrumental and solvent backgrounds, and scaled to a porous silica standard. The Porod invariant was evaluated from these measurements as in the observed q-range from 0.009 Å−1 < q < 0.06 Å−1. This quantity, Q*, is directly proportional to I(0) [I(0)/Q* = VP/2π2, where VP is the Porod volume), making it a useful metric for comparison to estimated values of I(0). (TIF) [file pbio.2002214.s002.tif]

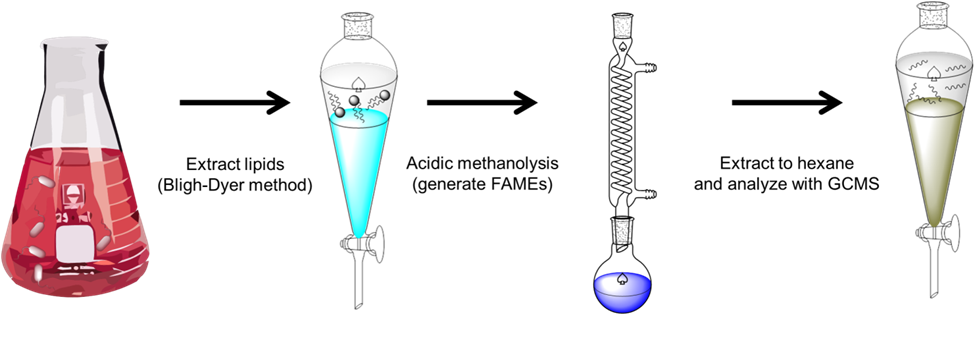

Supplement: S3 Fig — The total lipid fraction was extracted from an aliquot of cells using the Bligh-Dyer method. Following extraction, the lower lipid containing phase was dried under argon, and the lipids were heated in acidic methanol to create fatty acid methyl esters, which were then extracted using hexane and analyzed using GC/MS. (TIF) [file pbio.2002214.s003.tif]

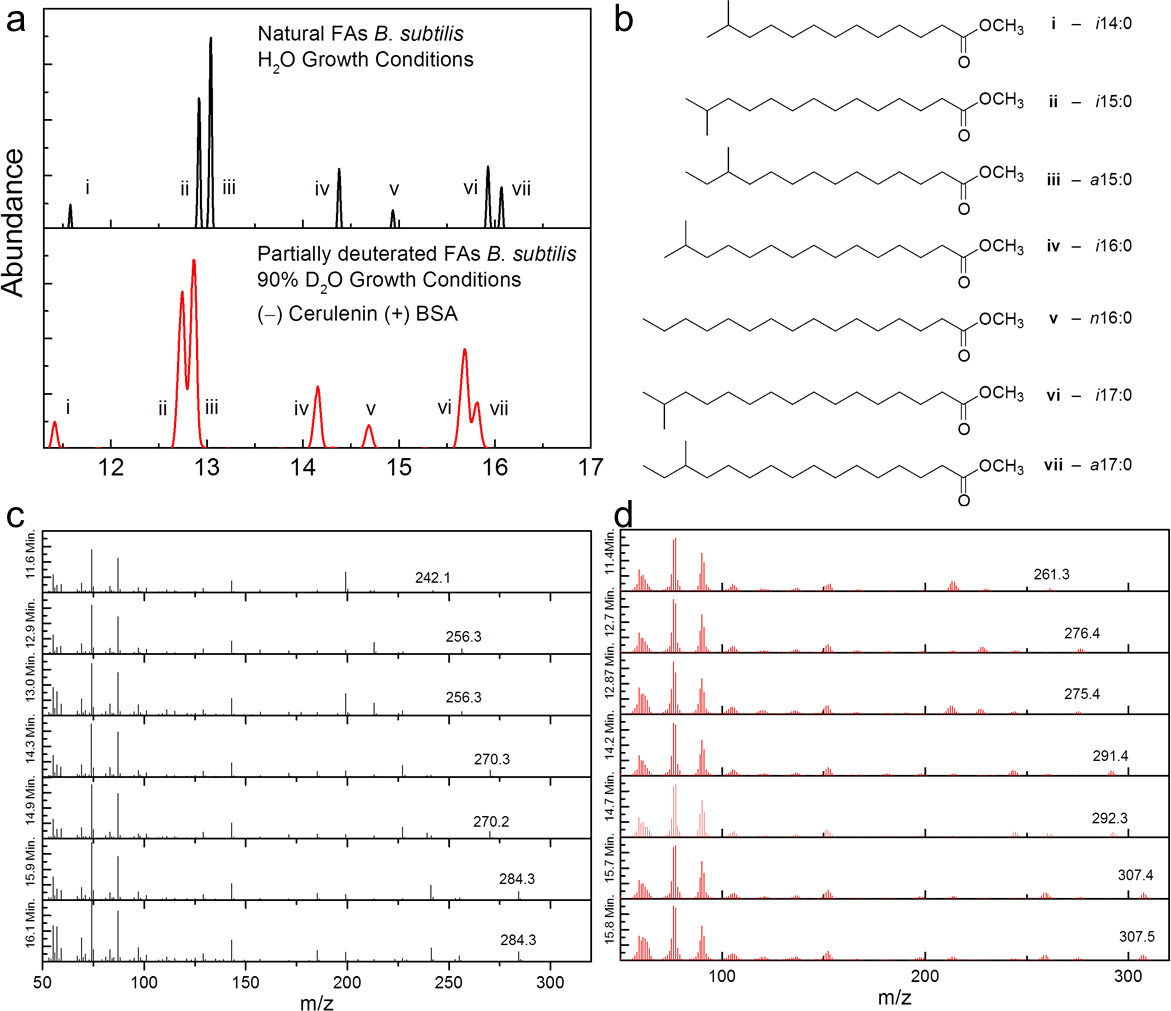

Supplement: S4 Fig — (a) GC/MS total ion chromatograms for FAMEs extracted from B. subtilis grown in M9 medium prepared with H-glucose and either H2O (black, top) or 90% D2O (red, bottom). The phospholipids in the native membrane of B. subtilis contain a mixture of saturated linear (normal) and branched chain (iso- or anteiso-) fatty acids shown in (b). As expected, 7 FAMEs were observed from cells cultured in 90% D2O (a, lower panel). Deuterated FAMEs eluted earlier, and their associated peaks were broader due to the presence of multiple isotopomers for each species. (c) and (d) show mass spectra for each FAME from cells grown in H- or D-medium, respectively. From the spectra, the extent of deuteration was determined by noting the change in mass of the molecular ion [M]+• (Table B in S1 Text and S2 Data). The distribution of isotopmers is shown in (d). (TIF) [file pbio.2002214.s004.tif]

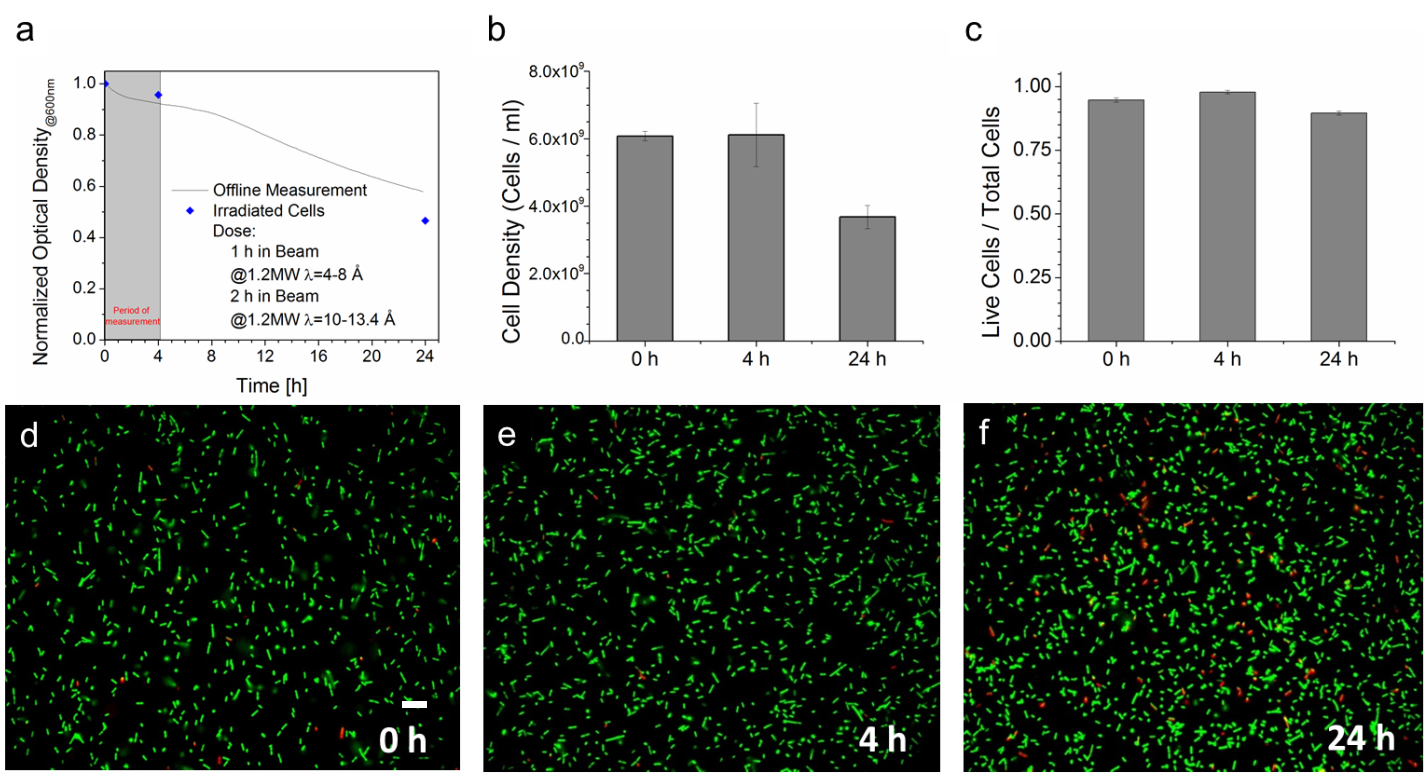

Supplement: S6 Fig — For SANS studies at 25°C, cells were suspended in 85% D2O PBS (pH 6.8) containing glucose (0.1% w/v), MgSO4 (10 mM), and cerulenin (50 μg/mL), and were then transferred to banjo-shaped quartz cuvettes with 1 or 2 mm beam paths. Once the cells were transferred, the cuvettes were sealed. SANS measurements were made over a maximum 4 h period. (a) OD600 drops off slowly over the first 8 h, and more rapidly thereafter. The blue diamonds denote OD600 measurements taken of an irradiated sample whose SANS spectrum is shown in Fig 4. The drop in OD600 over the 4 h measurement period was 5% for the sample in the beam and 7% for the control (non-irradiated), continuously monitored sample. (b) Cell densities were also determined through direct counts made using a hemocytometer on aliquots of the control sample from (a). Cell densities are consistent with OD600measurements. (c) The fraction of intact cells (which also happen to be alive) was quantified using a standard live/dead stain on aliquots of the continuously monitored sample from (a). Over the 24 h period of observation, >90% of intact cells stained green, indicating excellent cell viability and membrane integrity. After 4 h, which corresponds to maximum time that the cells were exposed to neutrons, 95% of the cells were alive (see S6 Data). (d-f) Representative false-colored, superimposed, red- and green-channel fluorescence micrographs corresponding to results shown in (c). Live cells appear green and dead cells appear red, the scale bars represents 20 μm. (TIF) [file pbio.2002214.s006.tif]

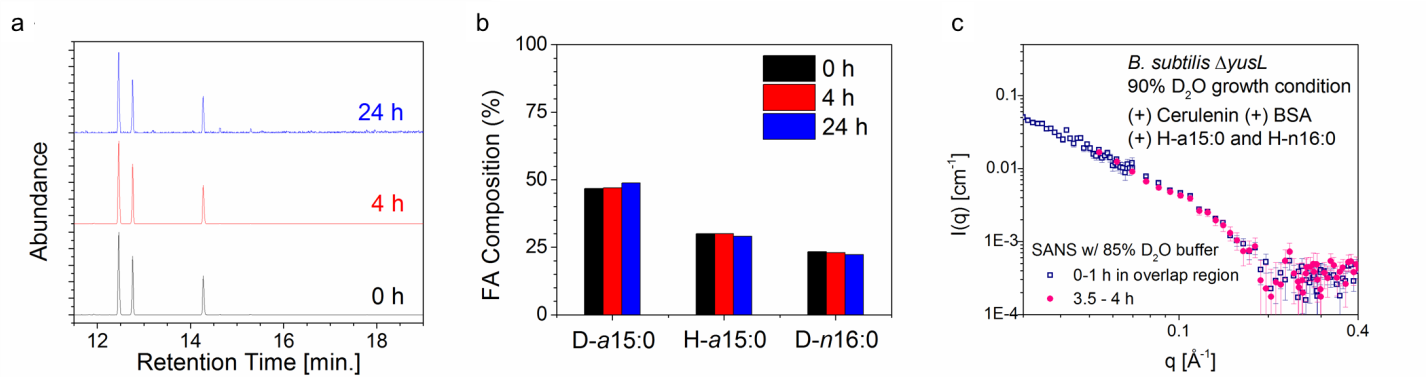

Supplement: S7 Fig — Fatty acid content over the period of the measurements was assessed by extracting the membrane lipids, performing an acidic methanolysis, and quantifying the FAME content using GC/MS. (a) Total ion chromatograms are shown for the lipids extracted from B. subtilis at harvest, after 4 hours of incubation–i.e., conditions which paralleled those of the SANS measurements–and after 24 h of incubation in modified PBS buffer. (b) Integrated peak areas for the chromatograms in (a). Less than 1% change is observed for any FA after 4 h, and only a 1–2% change after 24 h out of culture. (c) A repeat scattering measurement (pink) made 2 h after the initial measurement is superimposed onto the data shown in Fig 3 of the main text (blue). This scattering result shows that the sample is stable over the course of the 4 hour data collection period (see S6 Data). Note: the statistics of the repeat measurement are poorer (hence noise is greater), simply due to the shorter collection time. (TIF) [file pbio.2002214.s007.tif]

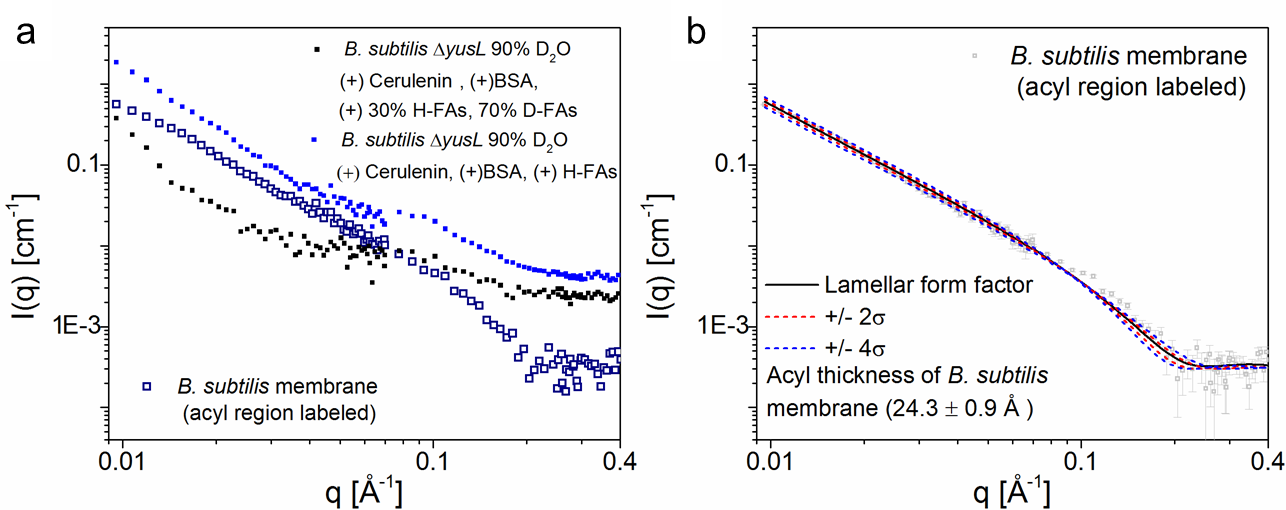

Supplement: S8 Fig — (a) The residual background was recorded using cerulenin-treated ΔyusL cells, which were fed a mixture of FAs contrast-matched to 85% D2O (a15:0 and n16:0, each 30% H and 70% D), sample scattering was recorded from cells cultured identically, except for being provided H-a15:0 and H-n16:0 in the culture medium. Subtraction of the background from the sample scattering revealed a pure membrane spectrum, which displayed a lamellar form factor characteristic of a lipid bilayer (Fig 3b). (b) Fitting of the lamellar form factor with fits illustrating the model envelope ± 2σ and ± 4σ superimposed on the data. (TIF) [file pbio.2002214.s008.tif]

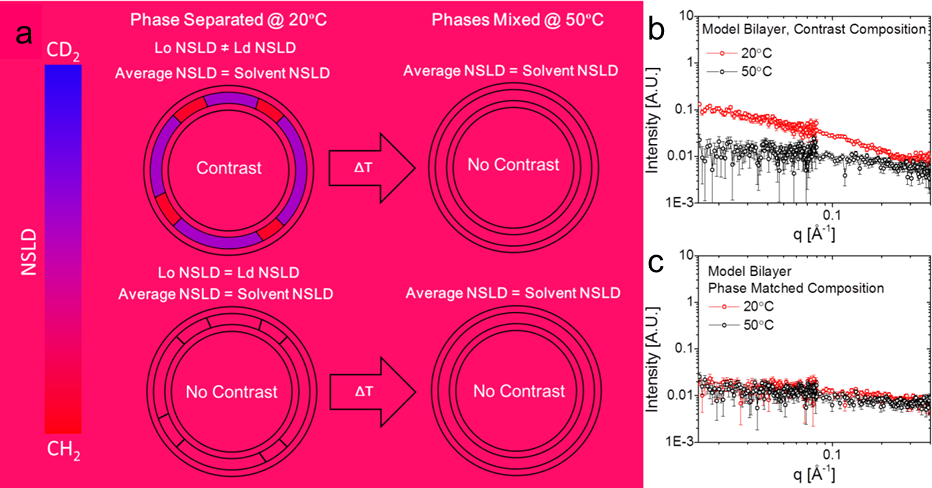

Supplement: S9 Fig — We demonstrate how neutron contrast can be manipulated by isotopic substitution to reveal lateral de-mixing using vesicles of POPC/DSPC/Chol. (see Table F in S1 Text and S7 Data). Detection of de-mixing requires observations of the bilayer in two states, i.e., mixed (pink) and de-mixed (red and blue). This can be accomplished using two strategies. The first (a, upper row) compares a system in two different states (i.e., mixed and de-mixed). At high temperature, the different NSLD lipids are uniformly mixed, with their average NSLD matching that of the buffer. Lowering the temperature causes the lipids to de-mix. Although the average lipid NSLD has not changed, the domains and surround have NSLDs which differ from the buffer’s NSLD and each other, resulting in excess scattering. We performed this experiment, and the results are shown in (b). Although this approach can be used successfully when temperature is a variable, it cannot be applied to live cells, as temperature changes can adversely affect their viability. Alternatively (a, lower row), one can construct a system where the domains and surround have the same average NSLD, which matches the buffer. This is achieved by providing all components at the same isotopic ratio, so that the same NSLD is kept whether or not the lipids are mixed or de-mixed. In this scenario, the scattering signal is the same for both states, as shown in (c). This strategy was adapted for the B. subtilis experiments at 25°C, where only isotopic substitution was used to manipulate contrast in the bilayer. (TIF) [file pbio.2002214.s009.tif]

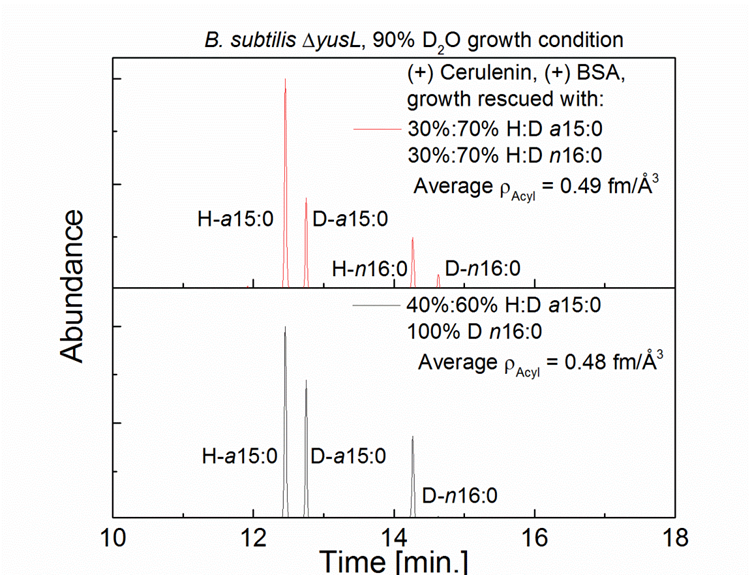

Supplement: S10 Fig — An aliquot from samples used in Fig 4 (main text) was analyzed for FA content. The GC/MS data show that the expected ratios of H- and D-FAs were incorporated in the cell’s membrane (see S8 Data). Peak areas are listed in Table E in S1 Text. (TIF) [file pbio.2002214.s010.tif]

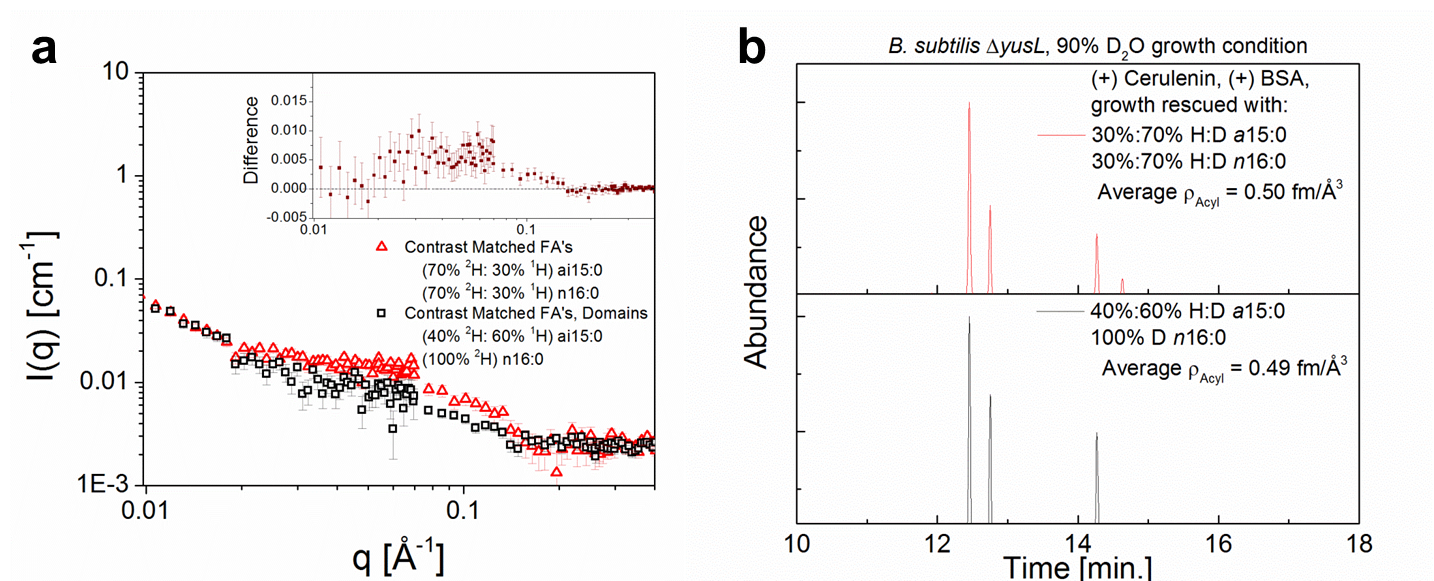

Supplement: S11 Fig — (a) The experiments shown in Fig 4 were repeated. The same excess scattering is observed in the q-range of ~0.015 to 0.15 Å−1, confirming the presence of nanoscopic features on the order of 40 nm. (b) GC/MS chromatograms for the lipids in these membranes (colors correspond to the spectra in panel (a), showing the expected H/D distribution of fatty acids, analogous to that shown in S10 Fig (see S8 Data). (TIF) [file pbio.2002214.s011.tif]
