# Supplementary material for: The in vivo structure of biological membranes and evidence for lipid domains
Source: PLoS Biol. 2017 May 23;15(5):e2002214. doi: 10.1371/journal.pbio.2002214 (PMC5441578; doi:10.1371/journal.pbio.2002214)
Supplement: S1 Text — (DOCX) [file pbio.2002214.s012.docx]

# The in vivo structure of biological membranes and evidence for lipid domains

Jonathan D. Nickels^1,2,3, ☯^, Sneha Chatterjee^2,4, ☯^, Christopher B. Stanley^2^, Shuo Qian^2^, Xiaolin Cheng^5,6^, Dean A.A. Myles^2^, Robert F. Standaert^1,2,6,*^, James G. Elkins^4,7,*^, and John Katsaras^1,2,3,*^

^1^Shull Wollan Center — a Joint Institute for Neutron Sciences, Oak Ridge National Laboratory Oak Ridge, Tennessee, United States

^2^Biology & Soft Matter Division, Oak Ridge National Laboratory, Oak Ridge, Tennessee, United States

^3^Department of Physics & Astronomy, University of Tennessee, Knoxville, Tennessee, United States

^4^Biosciences Division, Oak Ridge National Laboratory, Oak Ridge, Tennessee, United States

^5^Center for Molecular Biophysics, Oak Ridge National Laboratory, Oak Ridge, Tennessee, United States

^6^ Department of Biochemistry & Cellular and Molecular Biology, University of Tennessee, Knoxville, Tennessee, United States

^7^Department of Microbiology, University of Tennessee, Knoxville, Tennessee, United States

☯These authors contributed equally to this work.

^*^ [standaertrf@ornl.gov](mailto:standaertrf@ornl.gov) (RFS); [elkinsjg@ornl.gov](mailto:elkinsjg@ornl.gov) (JGE); [katsarasj@ornl.gov](mailto:katsarasj@ornl.gov) (JK)

**This Document Contains:**

**6 Supporting Tables**

**Table A. Abundances and selected physical properties of major cellular species.**

| Species | Mass %^a,b^ | Density^b^  (g/cm^3^) | Volume % | ρ_H_^c^  (fm/Å^3^) | Δρ_XD_^c^  (fm/Å^3^) | Δρ_CD_^c^  (fm/Å^3^) |
| --- | --- | --- | --- | --- | --- | --- |
| Protein^d^ | 12.99 | 1.39 | 9.92 | 0.190 | 0.137 | 0.472 |
| RNA^e^ | 4.41 | 1.76 | 2.67 | 0.345 | 0.106 | 0.262 |
| DNA^f^ | 0.64 | 1.67 | 0.41 | 0.318 | 0.066 | 0.317 |
| CHO^g^ | 0.69 | 1.43 | 0.51 | 0.181 | 0.132 | 0.440 |
| Lipid^h^ | 1.27 | 0.98 | 1.38 | –0.035 | 0.000 | 0.668 |
| Water | 80.00 | 1.00 | 85.11 | –0.056 | 0.696 | 0.000 |

a) Overall dry composition data from Bishop [1] were used with an assumed water content of 80% by weight.

b) Mass percentages and densities, which vary with deuteration, are reported for all-H species. Volume percentages were assumed to be invariant with deuteration.

c) ρ_H_ refers to the neutron scattering length density of all-H material. Δ*ρ* Refers to the change in *ρ* resulting from substitution of deuterium for all water-exchangeable (XD, X = N, O or S) or all water-nonexchangeable (CD) hydrogen atoms with deuterium, as denoted by the subscript. All neutron scattering lengths are from Sears [2] and were obtained through the NIST Center for Neutron Research (https://www.ncnr.nist.gov/resources/n-lengths/).

d) Residue volumes for amino acids were from Zamyatnin [3]. Density was calculated from residue volumes weighted by the amino acid composition in Table B.

e) The composition for *B. subtilis* RNA was from Midgley [4], and residue volumes were from Voss and Gerstein [5]. Density was calculated from the composition and residue volumes.

f) The G+C content of *B. subtilis* 168 DNA (43.5%) was from the genomic sequence [6], and residue volumes were from Nadassy et al. [7] Density was calculated from the composition and residue volumes.

g) Carbohydrate (CHO) was approximated as chitin, i.e., poly(*N*-acetylglucosamine) with an experimental density of 1.425 as reported by Li, et al [8].

h) Lipid is taken as the hydrocarbon portion of membrane fatty acids based on scattering length densities and composition given in Table B and Table D, respectively.

**Table B. Fatty acid composition and deuteration of samples in Figs 1 and 2. Additional information can be found in S3 Fig.**

| Fatty Acid | Relative Abundance H_2_O Medium | Relative Abundance 90% D_2_O Medium | FAME [M]^+•^ (m/z) H_2_O Medium | FAME [M]^+•^ (m/z) 90% D_2_O Medium | FA hydrogen atoms | Deuteration^a^ |
| --- | --- | --- | --- | --- | --- | --- |
| *i*14:0 | 4% | 3% | 242.1 | 261.3 | 27 | 71.1% |
| *i*15:0 | 24% | 26% | 256.3 | 276.4 | 29 | 69.3% |
| *a*15:0 | 35% | 30% | 256.3 | 275.4 | 29 | 65.9% |
| *i*16:0 | 12% | 10% | 270.3 | 291.4 | 31 | 68.1% |
| *n*16:0 | 4% | 4% | 270.2 | 292.3 | 31 | 71.3% |
| *i*17:0 | 13% | 19% | 284.3 | 307.4 | 33 | 70.0% |
| *a*17:0 | 9% | 8% | 284.3 | 307.5 | 33 | 70.3% |

a) Percent deuteration is calculated as 100*Δ(m/z) / FA hydrogen atoms, where Δ(m/z) is the increase in m/z for the molecular ion [M]^+•^ from growth in deuterated medium.

**Table C. Abundance and deuteration of cellular amino acids. (Notes on next page)**

| AA (abundance)^a^ | n_CH_^b^ | Fragment^c^ | Mass Range | Calc'd MW | Experimental Molecular Weights^d^ | | | *B. sub.* %D avg.^e^ |
| --- | --- | --- | --- | --- | --- | --- | --- | --- |
|  |  |  |  |  | H-Algal (error) | D-Algal (%D) | *B. Sub.* (%D) |  |
| Ala (11.97%) | 4 | M − C_5_H_9_O  M − C_4_H_9_ | 232–240  260–268 | 232.49  260.50 | 232.35 (-0.14)  260.35 (-0.15) | 236.27 (98.0)  264.29 (98.4) | 234.83 (62.0)  262.84 (62.2) | 62.1 |
| Arg (3.99%) | 7 |  |  |  |  |  |  | 64.1 (Glu) |
| Asn (3.71%) | 3 |  |  |  |  |  |  | 41.8 (Asp) |
| Asp (4.88%) | 3 | M − C_5_H_9_O  M − C_4_H_9_ | 390–397  418–425 | 390.76  418.77 | 390.71 (-0.05)  418.70 (-0.07) | 393.32 (86.8)  421.33 (87.5) | 391.96 (41.7)  419.96 (41.9) | 41.8 |
| Cys (1.21%) | 3 |  |  |  |  |  |  | 42.6 (Ser) |
| Gln (4.45%) | 5 |  |  |  |  |  |  | 64.1 (Glu) |
| Glu (8.43%) | 5 | M − C_7_H_15_O_2_Si  M − C_5_H_9_O  M − C_4_H_9_ | 330–339  404–413  432–441 | 330.63  404.79  432.80 | 330.55 (-0.09)  404.64 (-0.15)  432.69 (-0.11) | 335.15 (92.1)  409.31 (93.5)  437.31 (92.5) | 333.72 (63.5)  407.90 (65.1)  435.87 (63.7) | 64.1 |
| Gly (9.98%) | 2 | M − C_5_H_9_O  M − C_4_H_9_ | 218–224  246–252 | 218.46  246.47 | 218.34 (-0.13)  246.34 (-0.13) | 220.11 (88.5)  248.12 (88.9) | 219.72 (69.2)  247.74 (69.7) | 69.5 |
| His (1.73%) | 5 |  |  |  |  |  |  | 64.1 (Glu) |
| Ile (5.80%) | 10 | M − C_7_H_15_O_2_Si  M − C_5_H_9_O | 200–214  274–288 | 200.42  274.57 | 200.25 (-0.16)  274.37 (-0.20) | 210.07 (98.2)  284.21 (98.4) | 206.34 (60.9)  280.48 (61.1) | 61.0 |
| Leu (7.42%) | 10 | M − C_5_H_9_O | 274–288 | 274.57 | 274.37 (-0.20) | 284.25 (98.8) | 280.35 (59.8) | 59.8 |
| Lys (6.89%) | 9 |  |  |  |  |  |  | 60.0 (estimate) |
| Met (2.38%) | 8 | M − C_7_H_15_O_2_Si  M − C_5_H_9_O  M − C_4_H_9_ | 218–230  292–306  320–332 | 218.45  292.61  320.62 | 218.32 (-0.13)  292.45 (-0.16)  320.46 (-0.16) | 226.17 (98.1)  300.41 (99.6)  328.31 (98.1) | 222.78 (55.8)  297.01 (57.1)  324.95 (56.1) | 56.3 |
| Phe (3.69%) | 8 | M − C_5_H_9_O  M − C_4_H_9_ | 308–320  336–348 | 308.59  336.60 | 308.40 (-0.19)  336.41 (-0.19) | 316.29 (98.7)  344.31 (98.8) | 311.88 (43.4)  339.89 (43.5) | 43.5 |
| Pro (3.53%) |  |  |  |  |  |  |  | 64.1 (Glu) |
| Ser (4.82%) | 3 | M − C_7_H_15_O_2_Si  M − C_5_H_9_O  M − C_4_H_9_ | 288–295  362–369  390–397 | 288.60  362.75  390.76 | 288.39 (-0.21)  362.52 (-0.23)  390.53 (-0.23) | 291.32 (97.7)  365.44 (97.3)  393.46 (97.6) | 289.68 (43.0)  363.80 (42.6)  391.80 (42.3) | 42.6 |
| Thr (4.82%) | 5 | M − C_5_H_9_O  M − C_4_H_9_ | 376–385  404–413 | 376.78  404.79 | 376.48 (-0.29)  404.53 (-0.25) | 381.46 (99.5)  409.46 (98.6) | 379.94 (69.1)  407.97 (68.8) | 68.9 |
| Trp (1.03%) | 8 |  |  |  |  |  |  | 0.00 (added to medium) |
| Tyr (2.52%) | 7 |  |  |  |  |  |  | 43.5 (Phe) |
| Val (6.75%) | 8 | M − C_5_H_9_O  M − C_4_H_9_ | 260–272  288–300 | 260.54  288.55 | 260.36 (-0.18)  288.37 (-0.18) | 268.24 (98.5)  296.27 (98.7) | 264.63 (53.4)  292.66 (53.6) | 53.5 |

a) The experimental amino acid composition for *B. subtilis* was from Sauer et al. [9] The abundance of tryptophan along with the ratios for aspartate/asparagine (1.31) and glutamate/glutamaine (1.89) were not determined by Sauer et al., and the missing values were taken from the genomic average [10].

b) Number of skeletal hydrogens (C–H).

c) Parent compounds are bis(*tert*-butyldimethylsilyl) derivatives for Ala, Gly, Ile, Leu, Met, Phe and Val or tris(*tert*-butyldimethylsilyl) derivatives for Asp, Glu and Thr. Fragments used for molecular weight determination were selected from those validated for isotopomer analysis by Antoniewicz, et al. [11], and all retain the full complement of skeletal hydrogens.

d) Mass spectral data were recorded at unit resolution. Molecular weights *M_r_* were calculated as the average mass across the distribution of isotopomers, i.e., *M_r_* = Σ*_m_* *mI_m_ /* Σ*_m_* *I_m_*, where *I_m_* is the signal intensity, for all *m* in the indicated mass ranges. For the H-algal amino acid standards, the mass error (experimental – calculated) is reported. For deuterated amino acids, the extent of deuteration was calculated by substracting the molecular weight of the corresponding H-species from that of the deuterated species, and dividing the difference by the number of skeletal hydrogens.

e) Where an experimental value was unavailable, the value for a biosynthetic precursor (specified in parentheses) was used in most cases.

**Table D. Neutron scattering length and scattering length density calculated from atomic scattering lengths [2], isotopic content, and molecular volume [12].**

| Neutron Scattering Lengths (B) | | | | | | | | | |
| --- | --- | --- | --- | --- | --- | --- | --- | --- | --- |
| Element | | C | O | N | P | H | D |  |  |
| Coherent B (fm) | | 6.65 | 5.80 | 9.36 | 5.13 | -3.74 | 6.67 |  |  |
|  |  |  |  |  |  |  |  |  |  |
| Neutron Scattering Length Density Calculations, (ρ=B/Volume) | | | | | | | | | |
|  | Volume (Å^3^) | C | O | N | P | H | D | B (fm) | ρ (fm/Å^3^) |
| Solvent | | | | | | | | | |
| D_2_O | 30.4 | 0 | 1 | 0 | 0 | 0 | 2 | 19.15 | 0.63 |
| H_2_O | 30.4 | 0 | 1 | 0 | 0 | 2 | 0 | -1.68 | -0.06 |
| Hydrogenous Fatty Acids | | | | | | | | | |
| *i*14:0 | 408.5 | 13 | 0 | 0 | 0 | 27 | 0 | -14.6 | -0.04 |
| *i*15:0 | 436.4 | 14 | 0 | 0 | 0 | 29 | 0 | -15.4 | -0.04 |
| *a*15:0 | 436.4 | 14 | 0 | 0 | 0 | 29 | 0 | -15.4 | -0.04 |
| *i*16:0 | 464.3 | 15 | 0 | 0 | 0 | 31 | 0 | -16.3 | -0.04 |
| *n*16:0 | 444.2 | 15 | 0 | 0 | 0 | 31 | 0 | -16.3 | -0.04 |
| *i*17:0 | 492.2 | 16 | 0 | 0 | 0 | 33 | 0 | -17.1 | -0.03 |
| *a*17:0 | 492.2 | 16 | 0 | 0 | 0 | 33 | 0 | -17.1 | -0.03 |
| Deuterated Fatty Acids | | | | | | | | | |
| *a*15:0 | 436.4 | 14 | 0 | 0 | 0 | 0 | 29 | 286.5 | 0.66 |
| *n*16:0 | 444.2 | 15 | 0 | 0 | 0 | 0 | 31 | 306.5 | 0.69 |
| 70% Deuterated Fatty Acids | | | | | | | | | |
| *i*14:0 | 408.5 | 13 | 0 | 0 | 0 | 7.8 | 19.2 | 185.3 | 0.45 |
| *i*15:0 | 436.4 | 14 | 0 | 0 | 0 | 8.9 | 20.1 | 193.8 | 0.44 |
| *a*15:0 | 436.4 | 14 | 0 | 0 | 0 | 9.9 | 19.1 | 183.5 | 0.42 |
| *i*16:0 | 464.3 | 15 | 0 | 0 | 0 | 9.9 | 21.1 | 203.5 | 0.44 |
| *n*16:0 | 444.2 | 15 | 0 | 0 | 0 | 8.9 | 22.1 | 213.9 | 0.48 |
| *i*17:0 | 492.2 | 16 | 0 | 0 | 0 | 9.9 | 23.1 | 223.4 | 0.45 |
| *a*17:0 | 492.2 | 16 | 0 | 0 | 0 | 9.8 | 23.2 | 224.4 | 0.46 |

**Table E. Fatty acid composition and average ρ of fatty acids extracted from experimental conditions used in Figs 1–4. Values obtained by integration of GC/MS chromatograms shown in Figs 2c, S4 and S10.**

|  | *B. subtilis* 168 H_2_O M9 | *B. subtilis* Δ*yusL*  90% D_2_O M9 BSA | *B. subtilis* Δ*yusL* 90% D_2_O M9 BSA cerulenin *a*15:0 (0:100 D:H) *n*16:0 (0:100 D:H) | *B. subtilis* Δ*yusL* 90% D_2_O M9 BSA cerulenin *a*15:0 (70:30 D:H) *n*16:0 (70:30 D:H)  (Control Sample) | *B. subtilis* Δ*yusL* 90% D_2_O M9 BSA cerulenin *a*15:0 (60:40 D:H) *n*16:0 (100:0 D:H)  (Exptl. Sample) |
| --- | --- | --- | --- | --- | --- |
| Fig Nos. | 1, S3 | 2, S3 | 2, 3 | 4, S10 | 4, S10 |
| % H-Fatty Acids | | | | | |
| H-*i*14:0 | 3.8 | 0.0 | 0.0 | 0.0 | 0.0 |
| H-*i*15:0 | 23.9 | 0.0 | 0.0 | 0.0 | 0.0 |
| H-*a*15:0 | 35.2 | 0.0 | 84.0 | 20.2 | 26.0 |
| H-*i*16:0 | 11.5 | 0.0 | 0.0 | 0.0 | 0.0 |
| H-*n*16:0 | 3.7 | 0.0 | 16.0 | 4.1 | 0.0 |
| H-*i*17:0 | 12.9 | 0.0 | 0.0 | 0.0 | 0.0 |
| H-*a*17:0 | 8.9 | 0.0 | 0.0 | 0.0 | 0.0 |
| % Exogenous D-Fatty Acids | | | | | |
| D-*a*15:0 | 0.0 | 0.0 | 0.0 | 62.9 | 49.9 |
| D-*n*16:0 | 0.0 | 0.0 | 0.0 | 12.7 | 24.2 |
| % Endogenous (70% D) Fatty Acids | | | | | |
| *i*14:0 | 0.0 | 3 | 0.0 | 0.0 | 0.0 |
| *i*15:0 | 0.0 | 26 | 0.0 | 0.0 | 0.0 |
| *a*15:0 | 0.0 | 30 | 0.0 | 0.0 | 0.0 |
| *i*16:0 | 0.0 | 10 | 0.0 | 0.0 | 0.0 |
| *n*16:0 | 0.0 | 4 | 0.0 | 0.0 | 0.0 |
| *i*17:0 | 0.0 | 19 | 0.0 | 0.0 | 0.0 |
| *a*17:0 | 0.0 | 8 | 0.0 | 0.0 | 0.0 |
|  | | | | | |
| % Endogenous FAs | 100.0 | 100.0 | 0.0 | 0.0 | 0.0 |
| Average Acyl ρ (fm/Å^3^) | -0.03 | 0.44 | -0.03 | 0.5 | 0.49 |

**Table F. Experimental compositions and NSLD for proof of concept demonstration with model membranes, shown in Fig S9.**

| **Mole Fractions for POPC/DSCP/Chol. nanodomain forming lipid mixture [13,14,15]** | | | | | | | | |
| --- | --- | --- | --- | --- | --- | --- | --- | --- |
|  | | DSPC | | | POPC | | Chol | |
| Ld phase | | 0.09 | | | 0.79 | | 0.12 | |
| Lo phase | | 0.49 | | | 0.26 | | 0.25 | |
| Co-existing | | 0.39 | | | 0.39 | | 0.22 | |
| **Mole Fractions Used for Neutron Experiments** | | | | | | | | |
|  | DSPC | | d_70_-DSPC | POPC | | d_31_-POPC | Chol | d_41_-Chol |
| S9 Fig  upper panel (contrast if de-mixed) | 0.13 | | 0.26 | 0.39 | | 0.00 | 0.22 | 0.00 |
| S9 Fig  lower panel (uniform in all cases) | 0.31 | | 0.12 | 0.14 | | 0.25 | 0 | 0.22 |
| **Neutrons Scattering Length Density (fm/Å^3^)** | | | | | | | | |
|  | | Avg. SLD | | | Head Group SLD | | Acyl SLD | |
| Solvent (34.5% D_2_O) | | 0.18 | | |  | |  | |
| Ld phase (S9 Fig upper panel – de-mixed) | | 0.06 | | | 0.18 | | 0.02 | |
| Lo phase (S9 Fig upper panel – de-mixed) | | 0.23 | | | 0.18 | | 0.25 | |
| Avg. (mixed) (S9 Fig upper panel) | | 0.19 | | | 0.18 | | 0.19 | |
|  | | | | | | | | |
| Ld phase (S9 Fig lower panel – de-mixed) | | 0.19 | | | 0.18 | | 0.19 | |
| Lo phase (S9 Fig lower panel– de-mixed) | | 0.19 | | | 0.18 | | 0.20 | |
| Avg. (mixed) (S9 Fig lower panel) | | 0.19 | | | 0.18 | | 0.20 | |

**References:**

1. Bishop D, Rutberg L, Samuelsson B. The Chemical Composition of the Cytoplasmic Membrane of *Bacillus Subtilis*. Eur J Biochem. 1967;2(4):448–53.
2. Sears VF. Neutron Scattering Lengths and Cross Sections. Neutron News. 1992;3(3):26-37.
3. Zamyatnin A. Amino Acid, Peptide, and Protein Volume in Solution. Annu Rev Biophys Bioeng. 1984;13(1):145–65.
4. Midgley JEM. The Nucleotide Base Composition of Ribonucleic Acid from Several Microbial Species. Biochim Biophys Acta. 1962;61(4):513–25.
5. Voss N, Gerstein M. Calculation of Standard Atomic Volumes for Rna and Comparison with Proteins: Rna Is Packed More Tightly. J Mol Biol. 2005;346(2):477–92
6. Kunst F, Ogasawara N, Moszer I, Albertini AM, Alloni G, Azevedo V*, et al.* The Complete Genome Sequence of the Gram-Positive Bacterium *Bacillus Subtilis*. Nature. 1997;390(6657):249–56.
7. Nadassy K, Tomás-Oliveira I, Alberts I, Janin J, Wodak SJ. Standard Atomic Volumes in Double-Stranded DNA and Packing in Protein–DNA Interfaces. Nucleic Acids Res. 2001;29(16):3362–76.
8. Li J, Revol JF, Marchessault RH. Rheological Properties of Aqueous Suspensions of Chitin Crystallites. J Colloid Interface Sci. 1996;183(2):365–73.
9. Sauer U, Hatzimanikatis V, Hohmann H-P, Manneberg M, Van Loon A, Bailey JE. Physiology and Metabolic Fluxes of Wild-Type and Riboflavin-Producing *Bacillus Subtilis*. Appl Environ Microbiol. 1996;62(10):3687–96.
10. Moura A, Savageau MA, Alves R. Relative Amino Acid Composition Signatures of Organisms and Environments. PLoS ONE. 2013;8(10):e77319.
11. Antoniewicz MR, Kelleher JK, Stephanopoulos G. Accurate Assessment of Amino Acid Mass Isotopomer Distributions for Metabolic Flux Analysis. Anal Chem. 2007;79(19):7554–9.
12. Armen RS, Uitto OD, Feller SE. Phospholipid Component Volumes: Determination and Application to Bilayer Structure Calculations. Biophys J. 1998;75(2):734–44.
13. Heberle F, Petruzielo R, Pan J, Drazba P, Kučerka N, Standaert R, et al. Bilayer Thickness Mismatch Controls Domain Size in Model Membranes. J Am Chem Soc. 2013;135(18):6853–9.
14. Nickels JD, Cheng X, Mostofian B, Stanley C, Lindner B, Heberle FA, et al. Mechanical Properties of Nanoscopic Lipid Domains. J Am Chem Soc. 2015;137(50):15772–80.
15. Konyakhina TM, Goh SL, Amazon J, Heberle FA, Wu J, Feigenson GW. Control of a Nanoscopic-to-Macroscopic Transition: Modulated Phases in Four-Component DSPC/DOPC/POPC/Chol Giant Unilamellar Vesicles. Biophys J. 2011;101(2):L8–L10.
